# Supplementary figures and images for: nf-core/proteinfamilies: a scalable pipeline for the generation of protein families
Source: Gigascience. 2026 Jan 21;15:giag009. doi: 10.1093/gigascience/giag009 (PMC12950615; doi:10.1093/gigascience/giag009)

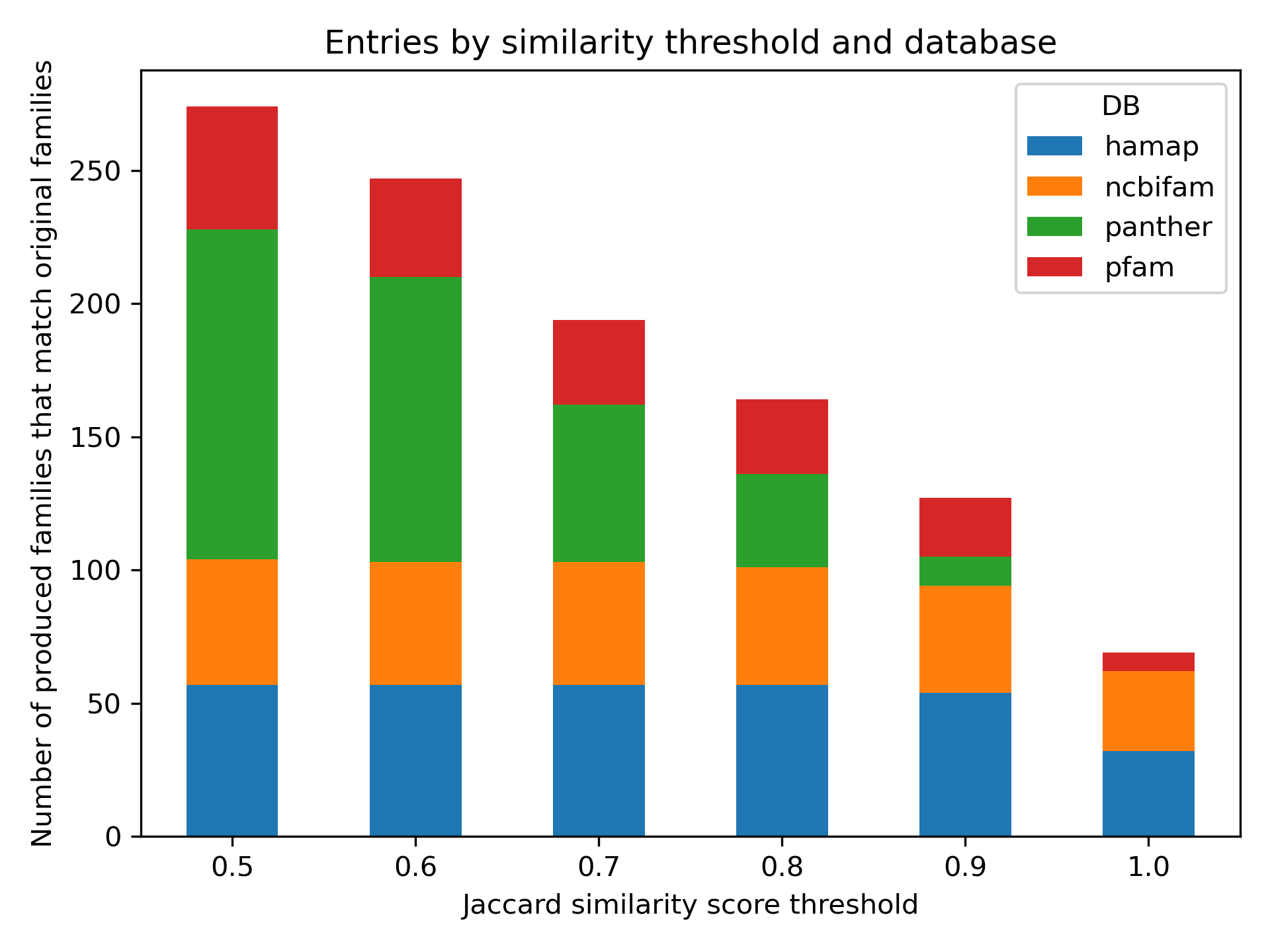

Supplement: giag009_Supplemental_Files [file giag009_supplemental_files.zip › Supplementary Figure 1.png]

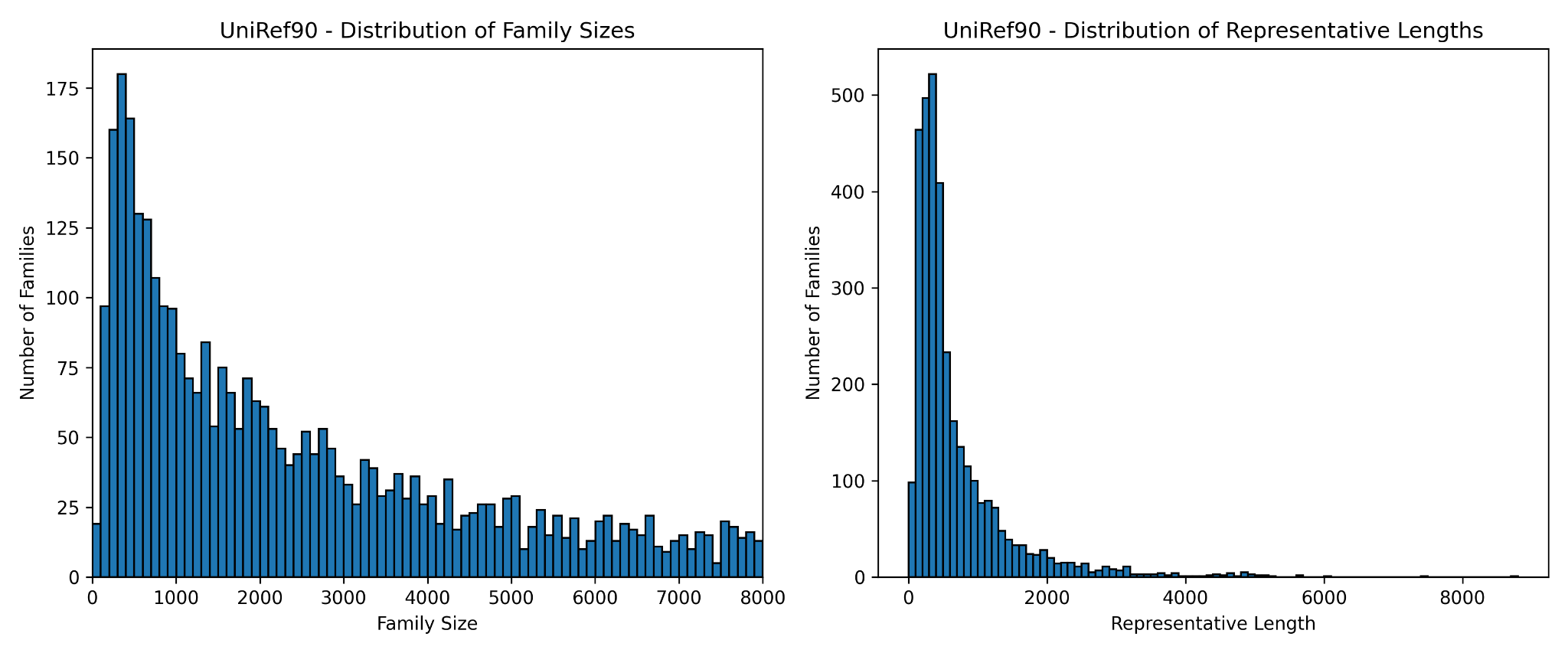

Supplement: giag009_Supplemental_Files [file giag009_supplemental_files.zip › Supplementary Figure 2.png]

UniRef90

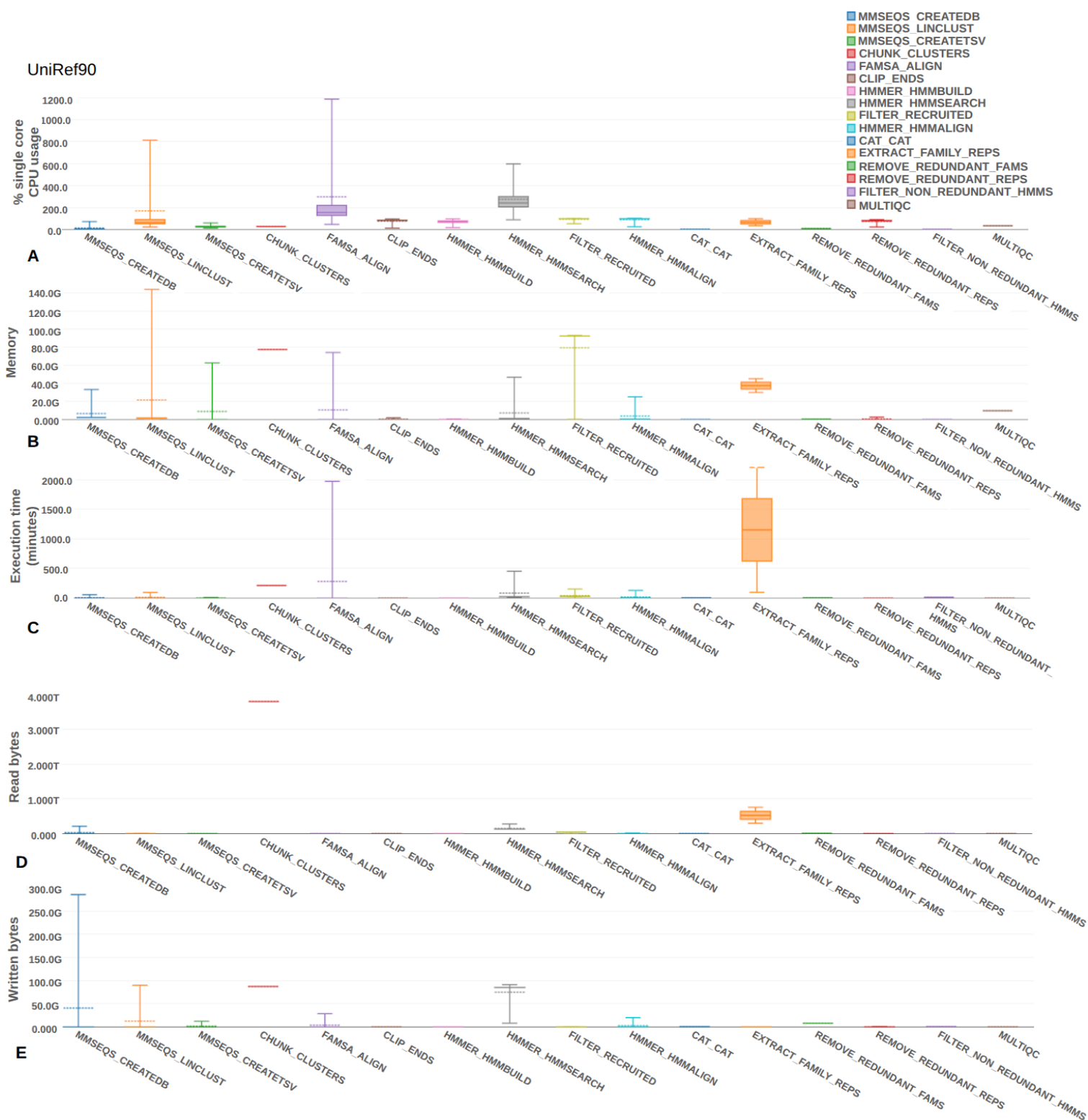

Supplement: giag009_Supplemental_Files [file giag009_supplemental_files.zip › Supplementary File 1.pdf]
