# Supplementary material for: nf-core/proteinfamilies: a scalable pipeline for the generation of protein families
Source: Gigascience. 2026 Jan 21;15:giag009. doi: 10.1093/gigascience/giag009 (PMC12950615; doi:10.1093/gigascience/giag009)
Supplement: giag009_GIGA-D-25-00328_Revision_1 [file giag009_giga-d-25-00328_revision_1.pdf]

GigaScience

nf-core/proteinfamilies: A scalable pipeline for the generation of protein families

--Manuscript Draft--

**Manuscript Number:**	GIGA-D-25-00328R1
**Full Title:**	nf-core/proteinfamilies: A scalable pipeline for the generation of protein families
**Article Type:**	Technical Note

Funding Information:

E.K. was funded from the European Union’s Horizon 2020 research and innovation program under the Marie Skłodowska-Curie grant agreement No 945405 (945405)	Dr Evangelos Karatzas
R.F, M.B.C and L.R were funded by EMBL	Dr Robert D. Finn
G.A.P. was supported by: Hellenic Foundation for Research and Innovation (H.F.R.I.) under the ‘Third Call for H.F.R.I. Research Projects to support faculty members and researchers’ [23592 - EMISSION] (23592)	Dr Georgios A. Pavlopoulos
F.A.B. was supported by: Hellenic Foundation for Research and Innovation (H.F.R.I.) under the ‘4th Call for H.F.R.I. Research Project to support Postdoctoral Researchers’ [28787-VIROMINE] (28787)	Dr Fotis A. Baltoumas
I.G.S. was supported by startup funds from the Penn State College of Medicine and the University of Texas at Austin	Dr Ilias Georgakopoulos-Soares
The work conducted by N.C.K. in the US Department of Energy Joint Genome Institute (https://ror.org/04xm1d337) was supported by the US Department of Energy Office of Science user facilities, operated under contract no. DE-AC02-05CH11231 (DE-AC02-05CH11231)	Dr Nikos Kyrpides

Abstract:

The growth of metagenomics-derived amino acid sequence data has transformed our understanding of protein function, microbial diversity, and evolutionary relationships. However, the vast majority of these proteins remain functionally uncharacterized. Grouping the millions of such uncharacterised sequences with the few experimentally characterised ones allows the transfer of annotations, while the inspection of conserved residues with multiple sequence alignments can provide clues to function, even in the absence of existing functional information. To address the challenges associated with this data surge and the need to group sequences, we present a scalable, open-source, parametrizable Nextflow pipeline (nf-core/proteinfamilies) that generates nascent protein families or assigns new proteins to existing families. The computational benchmarks demonstrated that resource usage scales approximately linearly with input size, and the biological benchmarks showed that the generated protein families closely resemble manually curated families in widely used databases.

**Corresponding Author:**	Evangelos Karatzas<br>EMBL-EBI: European Bioinformatics Institute<br>Hinxton, UNITED KINGDOM
**Corresponding Author Secondary Information:**	
**Corresponding Author's Institution:**	EMBL-EBI: European Bioinformatics Institute
**Corresponding Author's Secondary Institution:**	
**First Author:**	Evangelos Karatzas

Powered by Editorial Manager® and ProduXion Manager® from Aries Systems Corporation

**First Author Secondary Information:**	
**Order of Authors:**	Evangelos Karatzas
	Martin Beracochea
	Fotis A. Baltoumas
	Eleni Aplakidou
	Lorna Richardson
	James A. Fellows Yates
	Daniel Lundin
	- nf-core community
	Aydin Buluc
	Nikos Kyrpides
	Ilias Georgakopoulos-Soares
	Georgios A. Pavlopoulos
	Robert D. Finn
**Order of Authors Secondary Information:**	
**Response to Reviewers:**	We thank the editor and reviewers for the comments that helped us upgrade the quality of the pipeline and the manuscript. The main revision updates can be seen in the manuscript in red color. For further FAIR compliance, the following pipeline identifiers have been registered;<br>SciCrunch, RRID:SCR_027374 https://rrid.site/data/record/nlx_144509-1/RRID:SCR_027374/resolver?q=SCR_027374&i=rrid:scr_027374<br>workflowhub.eu https://workflowhub.eu/workflows/1954 latest version DOI: 10.48546/workflowhub.workflow.1954.3<br>bio.tools: https://bio.tools/nf-core_proteinfamilies<br><br>A point-by-point response to reviewer comments follows:<br><br>Reviewer #1<br>The authors present nf-core/proteinfamilies, a standardized Nextflow workflow that constructs protein families de novo or classifies sequences against existing families. Using a curated 200-family benchmark and a UniRef90-scale run, the authors show that the pipeline attains high recall with efficient runtimes. Given the ever-increasing size of sequence databases, this work is timely and fills a practical gap in reproducible, at-scale family curation; I expect it to be adopted widely by many research groups.<br><br>I have several comments and suggestions below:<br><br>1. In my view, this workflow will, by construction, yield a mixture of families: some anchored on a single conserved domain/segment, others centered on recurrent multi-domain cores, and some that capture the full-length sequence. This differs from widely used family databases: Pfam is largely domain-level, whereas HAMAP and NCBIFAM are mostly full-length/isofunctional (with PANTHER sitting in between). The resulting granularity is largely determined by MMseqs2 settings (sequence identity, query/target coverage, coverage mode) and by any alignment trimming, which biases toward conserved cores. Please add a brief discussion making this explicit, with practical guidance for tuning toward full-length versus domain-centric generation of families.<br><br>Reply:<br>We thank the reviewer for this comment. We agree that nf-core/proteinfamilies can produce a spectrum of family types (as found in Pfam), ranging from single domain families, to families containing multiple domains to full-length protein families. We have revised the manuscript to highlight the main parameters that can influence this granularity, offering practical guidance on how users can tune them to bias clustering toward domain-centric or full-length families, depending on the intended use case.

Powered by Editorial Manager® and ProduXion Manager® from Aries Systems Corporation

The “Parametrization” paragraph now reads:
Setting higher MMseqs2 coverage thresholds (e.g., ≥0.8) with alignment trimming enabled tends to bias protein family results toward conserved domains, whereas lower coverage thresholds (e.g., ≤0.5) with skipped trimming tend to produce more full-length or multi-domain families.

We also rephrased the relevant sentence in the discussion, which now reads:
Due to the nature of the initial seed generation and the strict sequence clustering coverage, our initial benchmark approach focused on matching conserved regions or domains rather than matching full-length proteins.

I also recommend a parameter-sensitivity analysis on the 200-family set: sequence identity (30-70%), coverage thresholds (50-95%), and coverage mode (query/target/both), with and without trimming. For each setting, report (i) total families and split/merge rates per curated family, and (ii) a simple granularity readout, the proportion classified as domain-anchored, multi-domain, or full-length. This would clarify how parameter choices drive family counts and domain/full-length centricity, and help readers select defaults aligned with their use case.

Reply:
This comment was very useful for setting the default clustering parameters in the nf-core/proteinfamilies pipeline configuration, and we thank the reviewer for this. Based on the suggested parameter options, we conducted a series of benchmarks using the same 200 original families described in the reproducibility paragraph of the paper.

The original benchmark was run with the linclust algorithm and with the following parameters:
cluster_seq_identity = 0.5, cluster_coverage = 0.9, cluster_cov_mode = 0, trim_msa = true
The cluster_cov_mode = 0 indicates that both strands were checked for coverage and that MMseqs2 is using the greedy set-cover clustering mode.

For the new combinations of suggested benchmarks, we first tried the same parameters with the cluster algorithm, since the number of sequences is medium, and execution speed can be sacrificed for more sensitive initial clusters. This halved the total number of initial clusters, increasing their size, and finally matched five additional families (178 instead of 173) from NCBIFAM and PANTHER due to fewer split families and initial sequences that did not cluster at all. With this in mind, we decided to continue the remaining benchmark combinations with the cluster algorithm.

All parameter combinations are listed in the new Supplementary Table 4, and the corresponding family-matching results are in Supplementary Table 5. The best results were achieved by the `broad_and_trim` combination (cluster_seq_identity = 0.3, cluster_coverage = 0.5, cluster_cov_mode = 0, trim_msa = true), with a total of 192/200 matched families, only failing to match 2 Pfam and 6 PANTHER families, which did not disappear, but were split into more than one of the generated families. The same configuration but without trimming, `lost` 14 additional PANTHER families, indicating that the sampled PANTHER families are probably domain-centric. The stricter coverage combinations lagged, mainly because they failed to produce enough initial clusters. This is somewhat expected because many of the protein families have been extensively iterated to represent a broader evolutionary range. Based on these benchmarks, we also tried another MMseqs2 coverage and clustering mode (greedy incremental) along with the best-scoring parameters. This resulted in the same number of matched families; however, it led to a small increase in the number of initial clusters and final families, thereby unnecessarily producing more groups and wasting more compute.

The reproducibility benchmark and discussion paragraphs have been updated accordingly.

2. In the results, the splits/misses are concentrated in Pfam/PANTHER, while HAMAP/NCBIFAM are much closer to one-to-one (HAMAP 50/50). This suggests the inflated family count is driven, in part, by the domain-centric portion of the benchmark

Powered by Editorial Manager® and ProduXion Manager® from Aries Systems Corporation

rather than the method itself. Please add a brief note in the Discussion to make this explicit.

Reply:
We thank the reviewer for this comment. According to the additional benchmarks we ran, HAMAP was the easiest to reproduce across all experiments. Following, NCBIFam was also replicated the most, except when the identity and coverage parameters became too strict (0.7 and 0.95, respectively), in which case only 29 of its 50 original families were matched. PANTHER and Pfam matched percentages were indeed more related to the clustering parameters. Both were best replicated when clustering thresholds were more relaxed. Only PANTHER was affected by the alignment trimming, and only during the broader clustering experiment; not trimming the alignments affected the results, matching 14 fewer original families. This indicates that the approach to use when replicating protein families may differ depending on the nature of the target protein family resource (domain-centric, full-length, isofunctional, etc.).

However, through reproducibility benchmarks, we identified default parameter values for which the nf-core/proteinfamilies pipeline appears to replicate the original families at best across all four tested resources. The Discussion paragraph has been updated accordingly and now reads: “A series of follow-up benchmarks with different clustering and trimming parameter combinations highlighted that the approach to use when replicating protein families may differ, depending on the nature of the target protein family resource (e.g., domain-centric, full-length/multi-domain, and/or isofunctional). However, we managed to identify parameter values for which the nf-core/proteinfamilies pipeline appears to replicate the original families at best across all four of the tested resources. This parameter combination consists of lower sequence-identity and coverage thresholds for clustering, along with trimming the gappy ends of the produced alignments, and managed to replicate 193 out of the 200 initially sampled families.”

3. Since AFDB has models for most UniProt entries, could these models be used as an orthogonal purity check of the generated families, e.g., map members to AFDB and ask whether they cluster to the same fold by TM-score/Foldseek (allowing full-length differences when the family is domain-anchored)?

Reply:
We thank the reviewer for this suggestion. We agree that AlphaFold DB models, combined with structural comparison tools such as Foldseek, provide a promising, orthogonal strategy for assessing family purity at the fold level. However, nf-core/proteinfamilies is designed as a sequence-based family-generation workflow and does not currently include structure-prediction or structure-clustering modules. These tasks are the focus of complementary pipelines within the nf-core community (e.g., nf-core/proteinfold), and our long-term goal is to support the chaining of these complementary pipelines. In fact, we implemented the required pipeline chaining (i.e., nf-core/proteinfamilies to nf-core/proteinfold) that automatically prepares a downstream input samplesheet for nf-core/proteinfold in the latest release version of nf-core/proteinfamilies (2.1.0). This pipeline chaining functionality enables seamless structure prediction of the generated family representative sequences and structural annotation with Foldseek, matching homologs from any desired structure database (e.g., AlphaFoldDB, PDB). We have added a note in the Discussion to clarify this scope. The paragraph now reads: “While nf-core/proteinfamilies is focused on sequence-based clustering, structural similarity methods can provide a complementary means of assessing family purity. The increasing coverage of AlphaFoldDB and tools such as Foldseek could be used to verify that family members share the same structural superfamily. The nf-core/proteinfold (https://github.com/nf-core/proteinfold) pipeline can predict 3D structures for family representative sequences from nf-core/proteinfamilies and then identify their structural homologs using Foldseek. We enable this functionality by automatically generating a samplesheet for use as input in the downstream nf-core/proteinfold pipeline”

4. HHsearch-based merging of divergent splits. In my view, and the authors note this, several curated families split simply because sequences are very divergent. An optional HHsearch (HMM-HMM) pass could merge these back: merge only at high

probability (≈≥95%) with reciprocal coverage of the shorter model (≥0.6). It would be useful to include this as an optional stage in the pipeline.

Reply:
We thank the reviewer for the comment. This turned out to be a very valuable addition to the pipeline. We decided to create a new subworkflow (i.e., merge_families) that would sit between the family redundancy and the sequence redundancy removal. It makes sense, as this step reduces code duplication by either filtering out redundant, or merging similar families, before proceeding to sequence removal and full MSA rebuilding. However, instead of using additional new modules from the HHsuite library, we decided to reuse the results of the existing hmmsearch module that was used to identify family-level redundancies (i.e., family reps against family models). We now provide a second, lower-length threshold parameter in the IDENTIFY_REDUNDANT_FAMS module, which also outputs the similarity scores between family representative sequences and family profile HMMs. Above a user-defined similarity threshold (e.g., 0.9, –should be below the redundancy threshold, which was 1 for our benchmarks), the seed alignments of these families may be merged. The family-generation subworkflow will then be re-triggered for these merged clusters (align/trim/build hmm/recruit sequences/generate full alignment). Testing this new subworkflow against our benchmarks, with a similarity score of 0.9, we generated 930 families (97 fewer than the best parameter combination –broad_and_trim) while also matching an additional (previously split) original family. We carried out more experiments with lower length similarity thresholds, but then the benchmark would start failing to match all 50 HAMAP families.

The “Methodology/Redundancy removal” paragraph has been updated to read:
“Achieving a lower matching length threshold (e.g., 90%) still indicates similarity between families. The pipeline can optionally merge the seed alignments for such families and proceed to generate updated profile HMMs and full alignments.”

The “Protein Family Reproducibility Benchmark” paragraph has also been updated to read:
“Finally, by allowing the merging of generated families within our best-scoring `broad_and_trim` benchmark, with an hmmsearch length similarity score threshold of 0.9, we managed to generate 930 total families (97 fewer families than without merging) while also matching an additional (previously split) original family, matching a total of 193 out of the 200 initially sampled families.”

5. Optional annotation of de novo families. I think it would be useful to add an annotation step that compares each de novo family (family HMM or MSA) against curated resources (Pfam, NCBIFAM, PANTHER/HAMAP).

Reply:
We thank the reviewer for this suggestion. Annotation of protein families against curated resources (Pfam, NCBIFAM, PANTHER, HAMAP, FunFams, etc.) is indeed an essential step in downstream analysis. However, we have intentionally scoped the nf-core/proteinfamilies pipeline to focus only on de novo family generation and updating. Incorporating annotation directly here would duplicate functionality that is under active development in other nf-core pipelines (e.g., nf-core/proteinannotator). Instead, we support chaining these workflows so that users can run annotation as a dedicated stage after family generation, for example, on the output fasta file containing all family representative sequences. We recently added the hmmsearch module in the nf-core/proteinannotator pipeline to annotate sequence domains with the latest Pfam release (https://github.com/nf-core/proteinannotator/pull/61) as well as with functional domains from FunFams (https://github.com/nf-core/proteinannotator/pull/62). We aim to expand the domain annotation databases to optionally include NCBIFAM, PANTHER, and HAMAP in the future. We have added a note in the Discussion to clarify this design decision and to highlight that nf-core/proteinfamilies outputs (MSAs, HMMs, representative sequences) are structured precisely to enable seamless integration with downstream analysis.

The paragraph now reads: “After protein families are generated, applying annotations from curated databases is an obvious next step. To keep the scope modular, nf-core/proteinfamilies focuses exclusively on protein family generation, while annotation

functionality is being developed in complementary pipelines such as nf-core/proteinannotator (https://github.com/nf-core/proteinannotator). We also enable the chaining of nf-core/proteinfamilies to nf-core/proteinannotator, by producing the respective downstream samplesheet with the family representative sequences.”

Immediately after this we also added two sentences, describing the cost benefits of running the nf-core/proteinfamilies before heavy-compute downstream analyses such as structural prediction and the application of functional annotations:
“It is also important to note that predicting structures and annotating the millions of sequences generated by metagenomics studies at scale would incur substantial computational cost. By running the nf-core/proteinfamilies pipeline in between, we enable the reduction of the search space for downstream analyses by highlighting a representative sequence per family, as structural features and functional annotations are expected to be largely transferable among family members.”

6. Could you briefly outline your expectations for how the pipeline handles transmembrane segments, coiled-coils, repeats, and IDRs, classes prone to over-splitting under MMseqs2 seeding and trimming due to short-motif signal, low complexity, and variable lengths?

Reply:
We thank the reviewer for raising this question. Certain sequence classes (transmembrane segments, coiled-coils, repeats, and intrinsically disordered regions) are known to generate short, low-complexity motif signals that bias profile building, often leading to overly sensitive models and large clusters. This issue is recognized in the design of fast search tools (for example, MMseqs2 uses TANTAN masking to mitigate such effects), and the ability to generate seed clusters for such edge cases is typically part of the tool's parametrization. An example benchmark output of the pipeline is the sampled original family PF02996 –a chaperone protein containing six coiled coils. Even though the proteins from this family could not cluster (<3 members) with the parameter combination of cluster_seq_identity = 0.5, cluster_coverage = 0.9, both for linclust and cluster algorithms (i.e., original_benchmarks –Supplementary Table 4–), tuning these parameters to 0.3 and 0.7, respectively (i.e., broad_and_trim), allowed the replication of this Prefoldin alpha-like protein family. Further use cases include the PF01032 and PF03169 families of transmembrane transporters, which also failed to cluster during the original_benchmarks. However, during the broad_and_trim benchmark, PF01032 was matched to a generated family, whereas the members of PF03169 were split across four generated families, and four PF03169 sequences were completely left out of the seed clusters. The rest of the seven non-matched families (i.e., split) include another transmembrane transporter (PTHR43653), three enzyme families (PTHR10030, PTHR21227, PTHR48077), one transferase (PTHR23072), one Translation initiation factor (PTHR43381), and one CRISPR-Cas (PF09704) protein family.

Added a paragraph in Discussion
“Certain sequence classes, such as transmembrane segments, coiled-coils, repeats, and intrinsically disordered regions are known to bias sequence seeding and profile building by generating short, low-complexity motifs. This behaviour has previously been documented and mitigated in search tools via masking and compositional corrections (e.g., MMseqs2; masking/TANTAN). As a result, the nf-core/proteinfamilies pipeline may produce fragmented families or leave such sequences unclustered, consistent with challenges observed in curated databases. While the current workflow does not include explicit handling of such cases, its modular design allows downstream integration with structure-based analyses and repeat/transmembrane/intrinsically disorder region annotation tools to further explore these effects.”

Reviewer #2
This manuscript presents a Nextflow pipeline (nf-core/proteinfamilies) for large-scale protein-family generation. Overall, I think the paper is well written and clear. The pipeline appears very useful, and the reported results show good performance in both family reproducibility and computational efficiency.

Powered by Editorial Manager® and ProduXion Manager® from Aries Systems Corporation

I have a few minor comments requesting additional details:

1) Does the quality-check step only compute statistics, or is it also used to filter/clean the input set? If so, please specify the criteria and whether filtered sequences are excluded downstream.

Reply:
We thank the reviewer for this comment. This comment inspired us to update the quality check (QC) subworkflow, which previously only generated statistics, to now also enable sequence preprocessing, so users do not have to do it manually/externally before running the nf-core/proteinfamilies pipeline. From pipeline release version 1.4.0 and beyond, the QC subworkflow now produces statistics of the input sequences, re-formats (i.e., to upper case, removing gaps) and validates them, filters by user-defined min and max length thresholds (defaults [30, 5000]), removes duplicate sequences by name, and finally produces another QC statistics report after pre-processing. The SeqFu library is used to produce the statistics and MultiQC web reports, while the SeqKit library is used for the pre-processing steps. The “Quality check and pre-processing” paragraph of the “Methodology” section have been updated accordingly.

2) Which MMseqs2 clustering mode is used (set cover, connected components, or greedy)? Can this be changed within the pipeline? If configurable, please indicate the relevant parameters.

Reply:
To clarify the point concerning configurability, we have added two sentences in the beginning of the parametrization paragraph, stating:

“Pipeline parameters can be directly specified in the nextflow.config file, or be overridden by either providing an additional configuration file or by directly setting them in the run command. Furthermore, any tool parameters not exposed as Nextflow variables can be set via the ext.args variable for each process in the modules.config file.”

Addressing the comment about the clustering mode, the nf-core/proteinfamilies pipeline uses MMseqs2 for initial sequence clustering. It exposes all relevant MMseqs2 parameters to the user, allowing the clustering mode to be set as desired. As the Reviewer indicates, in MMseqs2, the clustering mode can be set either directly via the --cluster-mode parameter or indirectly via the --cov-mode parameter, which automatically selects the most appropriate clustering mode for each case (which can also be overridden by the user). In the nf-core/proteinfamilies modules configuration file, we have set the --cov-mode argument to read the configurable cluster_cov_mode parameter (default value = 0) from the Nextflow configuration file, for both MMseqs2 algorithms (i.e., cluster, linclust). This implies a bidirectional sequence coverage, and the clustering mode is automatically set to the greedy set cover mode. To clarify this in the manuscript, we added the following sentences in the first paragraph of the parametrization section:

“The default bidirectional value for coverage mode (cluster_cov_mode = 0) automatically sets the MMseqs2 clustering to greedy set cover mode. However, users can override this parameter either indirectly, by changing the coverage mode, or directly, by setting the --cluster-mode argument in the modules configuration file.”

3) In the reproducibility benchmark, you use DIAMOND BLASTp to assess similarity between the initial sequence set for the selected families and additional Swiss-Prot sequences. Which sequence identity and alignment coverage (if any) thresholds were applied?

Reply:
We thank the reviewer for the comment. The default values of DIAMOND BLASTp have now been clarified in the manuscript: “>60% sequence identity, the tantan repeat masking algorithm for alignments, and e-value ≤ 0.001.”

4) Counts and coverage (p. 6): You state that "These 709 families captured 96.66% of the original unique sequence identifiers (103,385 out of 106,959).". However, a few

Powered by Editorial Manager® and ProduXion Manager® from Aries Systems Corporation

	lines above the final input set is reported as 169,605 unique protein sequences. Could you please clarify the initial number of sequences and the actual coverage after family generation and redundancy reduction?<br><br>Reply:<br>Thank you for catching this error. The number 169,605 resulted from a previous benchmark execution that was not then correctly updated. The correct numbers are 106,959 (116,959 including the additional UniProt sequences) and 5,826 produced seed clusters, not 2,947. The sequence coverage before family redundancy removal is 104,522 / 106,959 (97.72%), and 103,385 / 106,959 (96.66%) after redundancy removal. The manuscript has now been updated appropriately, and we apologise for this oversight.<br><br>5) Figures S1 and S2 are challenging to read due to low resolution.<br><br>Reply:<br>We thank the reviewer for pointing this out. The S1 and S2 figures have now been updated to high resolution.
Additional Information:	
Question	Response
Are you submitting this manuscript to a special series or article collection?	No
**Experimental design and statistics**<br><br>Full details of the experimental design and statistical methods used should be given in the Methods section, as detailed in our Minimum Standards Reporting Checklist. Information essential to interpreting the data presented should be made available in the figure legends.<br><br>Have you included all the information requested in your manuscript?	Yes
**Resources**<br><br>A description of all resources used, including antibodies, cell lines, animals and software tools, with enough information to allow them to be uniquely identified, should be included in the Methods section. Authors are strongly encouraged to cite Research Resource Identifiers (RRIDs) for antibodies, model organisms and tools, where possible.<br><br>Have you included the information requested as detailed in our Minimum	Yes

Standards Reporting Checklist?	
**Availability of data and materials**<br><br>All datasets and code on which the conclusions of the paper rely must be either included in your submission or deposited in publicly available repositories (where available and ethically appropriate), referencing such data using a unique identifier in the references and in the "Availability of Data and Materials" section of your manuscript.<br><br>Have you have met the above requirement as detailed in our Minimum Standards Reporting Checklist?	Yes
GigaScience has policies and guidelines in place for the use of generative AI-writing tools such as ChatGPT. If you have used such writing tools to assist with writing the manuscript this must be declared and cited in the text. Authors should not list AI-writing tools and other AI-assisted technologies as an author or co-author and should acknowledge that they are fully responsible for text generated or refined by AI-writing tools.<p><br><br>A summary of use (particularly in the introduction or among methods) needs to be included at the end of the paper, and the outputs should also be included as a supplementary file hosted in GigaDB or other open repositories. Please read our guidelines for more information. <p><br><br>By submitting to GigaScience, you are aware of the journal's AI-writing tools policy, and if you have declared use of such tools below, you have acknowledged this where appropriate in your manuscript and have made a summary of use and outputs available. <p><br><b>AI-assisted writing tools have been used in the preparation of this manuscript?	No

Manuscript
Click here to access/download;Manuscript;revised_nf-core_proteinfamilies.docx

nf-core/proteinfamilies: A scalable pipeline for the generation of protein families

Evangelos Karatzas[1], Martin Beracochea[1], Fotis A. Baltoumas[2], Eleni Aplakidou[2,3], Lorna Richardson[1], James A. Fellows Yates[4], Daniel Lundin[5,6], nf-core community[7], Aydin Buluç[8,9], Nikos C. Kyrpides[10], Ilias Georgakopoulos-Soares[11,12], Georgios A. Pavlopoulos[2,13], Robert D. Finn[1,*]

[1] European Molecular Biology Laboratory, European Bioinformatics Institute (EMBL-EBI), Wellcome Genome Campus, Hinxton, Cambridge, UK.
[2] Institute for Fundamental Biomedical Research, BSRC "Alexander Fleming", Vari 16672, Greece.
[3] Division of Basic Sciences, University of Crete Medical School, Heraklion 71110, Greece
[4] Department of Archaeogenetics, Max Planck Institute for Evolutionary Anthropology, 04103 Leipzig, Germany
[5] Centre for Ecology and Evolution in Microbial Model Systems - EEMiS Linnaeus University, SE-39182 Kalmar, Sweden
[6] Dept. of Biochemistry and Biophysics, Stockholm University, SE-10691 Stockholm, Sweden
[7] https://nf-co.re
[8] Computational Research Division, Lawrence Berkeley National Laboratory, Berkeley, CA, USA.
[9] Department of Electrical Engineering and Computer Sciences, University of California, Berkeley, CA, USA.
[10] DOE Joint Genome Institute, Lawrence Berkeley National Laboratory, Berkeley, CA, USA.
[11] Division of Pharmacology and Toxicology, College of Pharmacy, The University of Texas at Austin, Dell Paediatric Research Institute, Austin, TX, USA.
[12] Institute for Personalized Medicine, Department of Biochemistry and Molecular Biology, The Pennsylvania State University College of Medicine, Hershey, PA, USA.
[13] Department of Computational Biology, Mohamed bin Zayed University of Artificial Intelligence (MBZUAI), Abu Dhabi, United Arab Emirates.

[*] Corresponding author: rdf@ebi.ac.uk

Abstract

The growth of metagenomics-derived amino acid sequence data has transformed our understanding of protein function, microbial diversity, and evolutionary relationships. However, the vast majority of these proteins remain functionally uncharacterized. Grouping the millions of such uncharacterised sequences with the few experimentally characterised ones allows the transfer of annotations, while the inspection of conserved residues with multiple sequence alignments can provide clues to function, even in the absence of existing functional information. To address the challenges associated with this data surge and the need to group sequences, we present a scalable, open-source, parametrizable Nextflow pipeline (*nf-core/proteinfamilies*) that generates nascent protein families or assigns new proteins to existing families. The computational benchmarks demonstrated that resource usage scales approximately linearly with input size, and

the biological benchmarks showed that the generated protein families closely resemble manually curated families in widely used databases.

Introduction

The generation of protein families is a common approach for facilitating the transfer of functional annotations from sequences with a determined function to uncharacterized sequences. Given the enormous volume of unannotated sequences obtained from metagenomic analyses, it is necessary to group sequences to transfer these functional annotations at scale [1,2]. Grouping related sequences and building protein family models can enable the detection and annotation of distant homologs across large datasets. Profile Hidden Markov Models (HMMs) are especially effective for this task, as they capture conserved sequence patterns and model insertions and deletions. Efficiently transferring annotations at scale can expedite key applications such as identifying novel enzymes for industrial use, predicting drug targets in pathogenic organisms, and reconstructing metabolic pathways in microbial communities. To achieve this, biological databases and platforms provide curated protein information and metadata to support both functional and 3D-structure annotation of protein families. Primary resources include UniProtKB [3], Protein Data Bank (PDB) [4], RefSeq [5], GenBank [6], IMG/M [7], Big Fantastic Database (BFD) [8], and MGnify [9]. These databases collectively host hundreds of millions to billions of sequences, many of which remain uncharacterized, particularly those derived from metagenomic sources.

While the aforementioned databases catalog raw sequence and structure data, protein classification resources help interpret this information by organizing proteins into families based on shared evolutionary or functional traits. Some widely used resources include Pfam [10], InterPro [11], the Novel Metagenome Protein Families Database (NMPFamsDB) [12,13], FunFams [14], eggNOG [15,16], KEGG Orthology (KO) [17], Clusters of Orthologous Groups (COG) [18] and its extension for eukaryotic proteins, KOG [19], each serving distinct purposes in protein classification. Notably, InterPro integrates protein family, domain, and functional site data from thirteen contributing databases. As of May 2025, InterPro provides standardized annotations for over 200 million proteins. Conversely, NMPFamsDB focuses on protein families derived from metagenomic and metatranscriptomic data that do not correspond to known reference genome proteins or Pfam domains. Its latest version features over 106,000 distinct protein families, each containing at least 100 sequences, cumulating to ~20 million proteins.

As the number of protein sequences grows, the manual curation of protein families at scale seems unfeasible. Computational methods for automatically generating protein families must therefore strike a balance between sensitivity, scalability, and usability. Pfam-B [20], a compendium to profile HMM-based families in Pfam, is a computationally generated set of putative protein families that complement the Pfam families. Pfam-B families are based on a bidirectional MMseqs2 clustering [21] – a high-speed tool for sequence clustering – and are recalculated at each Pfam major release, but are not readily transferable to other sequences. Another widely used clustering tool is CD-HIT [22], which, similarly to MMseqs2, prioritizes speed by employing greedy incremental clustering starting from longer sequences. However, both MMseqs2 and CD-HIT sacrifice sensitivity compared to profile HMM methods and pose challenges when transferring family annotations to additional sequences. Thus, there is a need

for streamlined, scalable solutions that can generate and maintain protein families from large, ever-expanding sequence datasets.

We have developed the *nf-core/proteinfamilies* pipeline to address many of the challenges mentioned above, by chaining modules for clustering sequences, generating protein family-level models, alignments, and metadata, removing redundancies, and updating families as more sequences become available. The pipeline leverages the Nextflow [23] workflow orchestrator and nf-core principles [24,25], enabling standardized pipeline execution, efficient resource management, and parallel processing, hence allowing users to handle large datasets effectively.

Methodology

The *nf-core/proteinfamilies* pipeline is a bioinformatics tool designed to generate new protein families or update existing ones, given a FASTA file of amino acid sequences as input, along with profile HMMs and alignments of existing families when updating. The main components of the pipeline are *(i) input sequences* quality check and pre-processing, *(ii)* optional model and alignment updating of existing families, *(iii)* sequence clustering, *(iv)* family generation, and *(v)* optional redundancy removal (Figure 1).

Quality check and pre-processing: The first stage employs a basic sequence quality check mechanism using SeqFu [26], which generates a report summarizing statistics such as the number of sequences, total amino acid count, and the minimum, average, and maximum sequence lengths. The SeqKit [27] library is also used to reformat (i.e., convert to uppercase and remove gaps) and validate input sequences, to filter sequences by user-defined minimum and maximum length thresholds (defaults [30, 5000]), and to remove duplicate sequences by name.

Existing families update: In this complementary workflow, users can enrich existing families with new sequences by recruiting new members into the full alignment of a family. When users provide existing family profile HMMs and multiple-sequence alignment (MSA) files – in compressed archive format (.tar.gz) – along with an amino acid sequence file, the pipeline begins by searching for matches of the input sequences against the current families. This is done by combining the existing HMMs into a library and performing an *hmmsearch* against that with the input sequences. For each family hit, the relevant input sequences are combined with the non-aligned sequences from the corresponding family MSA in an aggregated FASTA file, which can optionally undergo strict clustering to eliminate redundancies. The remaining sequences are then aligned and, optionally, undergo gap removal, resulting in an updated family full MSA. Finally, the family profile HMM is retrained based on the updated MSA.

Sequence clustering: The first stage of the workflow clusters protein sequences using MMseqs2, enabling fast clustering. The input FASTA file is converted into an MMseqs2 database, and then the MMseqs2 clustering algorithm of choice (*linclust* –focus on speed*, or cluster* –focus on sensitivity) is applied to group similar sequences into initial *seed* clusters. The clustering results are saved as a two-column (i.e., cluster representative, cluster member) TSV-formatted output, and are then filtered based on minimum membership.

Family generation: The next stage of the workflow generates protein families for each filtered cluster. In parallel, for each *seed* cluster, a multiple-sequence alignment is performed using either *FAMSA* [28] or *MAFFT* [29]. This process generates the seed MSA file of a family. An optional step allows users to trim poorly aligned or gap-rich regions from the seed MSA using *ClipKIT* [30], either across the entire alignment or only at the ends, thereby improving alignment quality for downstream analyses. The seed alignments are then used to generate profile HMMs via the *HMMER* (v3.4) *hmmbuild* command [31]. The *hmmsearch* command is optionally used to recruit additional sequences from the input FASTA file into the generated families, according to user-defined thresholds such as *e*-value or minimum matching HMM length. Subsequently, recruited sequences are realigned to the family HMM with the *hmmalign* tool, generating a full MSA file for each family.

Redundancy removal: In most cases, the initial MMseqs2 clustering does not assign all related sequences to a single cluster, leading to multiple clusters that may be evolutionarily related. In subsequent pipeline steps, the same sequence may be assigned to multiple clusters, resulting in multiple representations of the same protein family. Furthermore, in larger sequence datasets, the presence of identical or highly similar sequences within a family can unnecessarily consume storage space without contributing additional functional, evolutionary, or structural variability. To address these issues, the third stage of the pipeline employs two distinct mechanisms for redundancy detection and elimination. These mechanisms can be deployed both *(i) inter-family,* where redundant families are removed and/or similar families are merged, and *(ii) intra-family*, where redundant sequences (those with very similar identity and alignment coverage) within a family are eliminated. Users can choose to use both mechanisms together, separately, or skip this step.

The inter-family redundancy removal process involves several steps. First, all generated profile HMMs are combined into a single file to form a profile HMM library. Next, family representative sequences are searched against the profile HMM library using *hmmsearch*. The premise is that if a family representative sequence matches another family profile HMM with a strict matching length threshold (e.g., 100%), it suggests redundancy between the two families, allowing one to be labeled as redundant (currently removing the family with the fewest sequences). Achieving a lower matching length threshold (e.g., 90%) still indicates similarity between families. The pipeline can optionally merge the seed alignments for such families and proceed to generate updated profile HMMs and full alignments.

In the intra-family redundancy removal mechanism, the pipeline begins with stringent MMseqs2-based clustering of members (*default parameters: 0.9 identity, 0.9 coverage length, bidirectional*), retaining only the cluster representatives. This step removes duplicate or nearly identical sequences, reducing the family size while preserving a subset of diverse sequences that represent the family.

The workflow finishes with the generation of reports, including the input sequence quality check before and after pre-processing, the size distribution of the initial MMseqs2-generated clusters, and the produced family data and metadata using *MultiQC* [32]. The size distribution section reports the number of initial clusters for each observed size (number of proteins). The produced family report contains information such as unique family identifiers, family sizes, family

representative sequences, and their lengths. These results are presented in an interactive HTML report that provides users with a concise overview of the generated data.

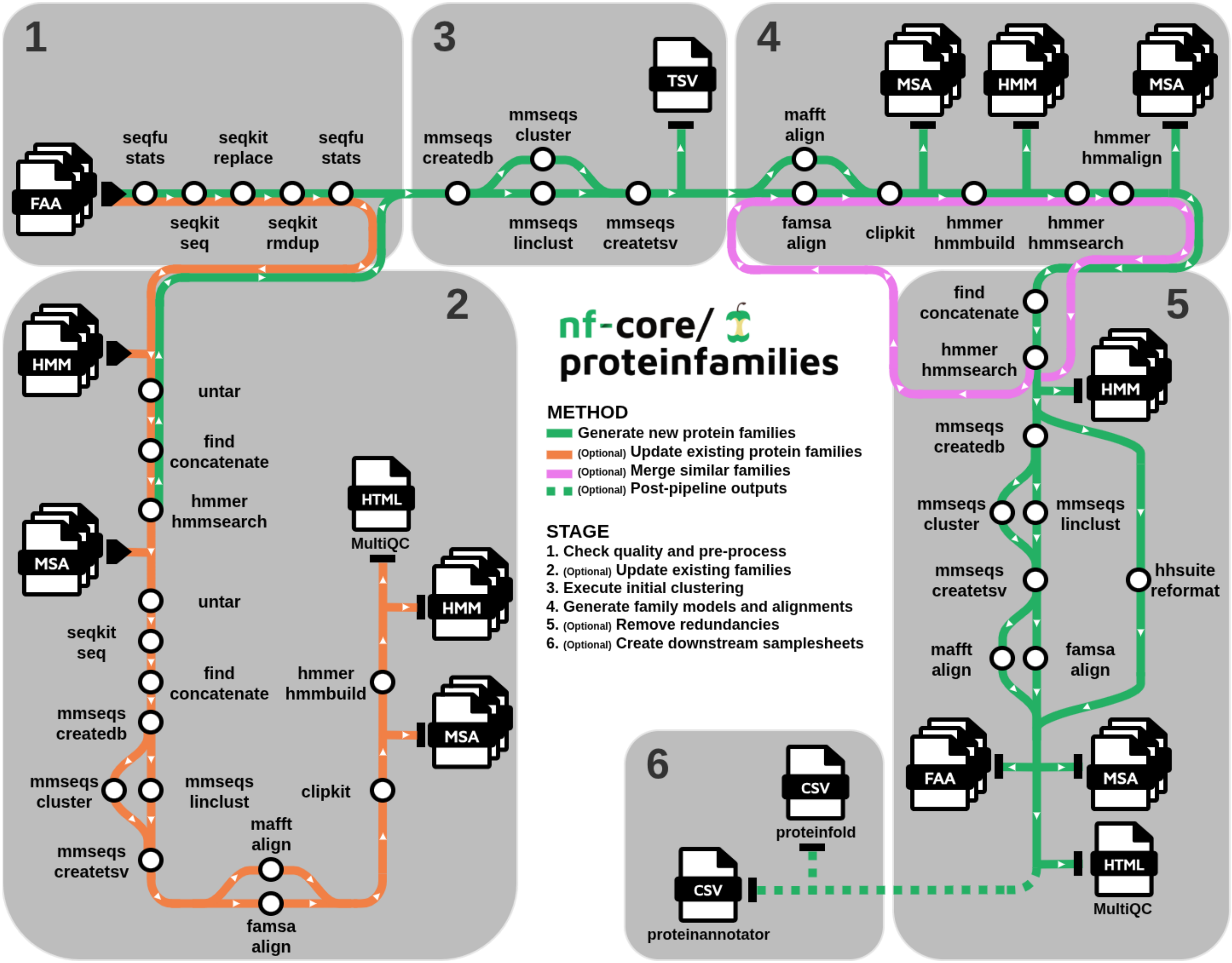

Figure 1. Workflow of the *nf-core/proteinfamilies* pipeline. The process begins with a mandatory input amino acid FASTA file, which undergoes a quality check using SeqFu and SeqKit (1) to generate sequence quality statistics, preprocess and validate the initial sequences, and remove duplicates. Optionally, in the *update existing families* stage (2), users can provide compressed archives containing HMMs and MSAs of existing families. Sequences that match existing families are integrated into those families, updating the respective MSAs and HMMs. Sequences without matches follow the default workflow (green path) to generate new families, starting with an initial MMseqs2 clustering (3) and then proceeding to family generation (4). Users may then optionally apply redundancy reduction (5), either across families (inter-family removal and/or merging) or within families (intra-family), to remove similar families or sequences, respectively. Finally, users can opt to produce samplesheets (6) for downstream analyses with other nf-core pipelines (i.e., protein folding and annotation).

Parameterization

Various user-defined parameters guide the execution flow. Pipeline parameters can be directly specified in the *nextflow.config* file, or be overridden by either providing an additional configuration file or by directly setting them in the run command. Furthermore, any tool parameters not exposed

as Nextflow variables can be set via the ext.args variable for each process in the *modules.config* file. For clustering, users can choose between one of two MMseqs2 clustering algorithms: *(i)* the standard '*cluster*' method (default), suitable for medium-sized inputs (thousands to a few million sequences), or *(ii)* '*linclust*', which offers faster but less sensitive clustering for larger sequence datasets (thousands of millions to a few billion sequences). Additional clustering parameters include sequence identity, query/target coverage, and coverage mode (unidirectional or bidirectional), which can be configured separately for the initial input sequence clustering and for the redundancy removal (strict clustering within families) steps. The default bidirectional value for coverage mode (cluster_cov_mode = 0) automatically sets the MMseqs2 clustering to *greedy set cover* mode. However, users can override this parameter either indirectly, by changing the coverage mode, or directly, by setting the --cluster-mode argument in the modules configuration file. Users can also define the minimum cluster size required for the downstream family generation.

There are two available multiple sequence alignment (MSA) algorithms: *(i)* FAMSA and *(ii)* MAFFT. FAMSA is the default option due to its balance of time efficiency, memory usage, and overall accuracy, while MAFFT can still outperform it in certain edge cases [33]. Users may trim MSA columns that exceed a defined gap percentage threshold using ClipKit, either along its entire length or only at its ends. Setting higher MMseqs2 coverage thresholds (e.g., ≥0.8) with alignment trimming enabled tends to bias protein family results toward conserved domains, whereas lower coverage thresholds (e.g., ≤0.5) with skipped trimming tend to produce more full-length or multi-domain families. To expand sequence families, users are encouraged to search the input sequences against the initial family profile HMMs generated by seed MSAs. This can be achieved by using *HMMER/hmmsearch*, where users can specify an e-value cutoff and minimum length threshold.

Lastly, users may enable redundancy removal after families are constructed. This process is controlled by two settings: one that eliminates redundancy between families, either by removing identical or merging highly similar families, and another that removes redundancy within families. Enabling both options, which is the default in the pipeline, is recommended to prevent family and sequence duplication while preserving sequence diversity. The amino acid sequence files (FASTA) generated throughout the pipeline can also be saved in the results folder by setting the appropriate parameters.

Protein Family Reproducibility Benchmark

To evaluate the applicability of the pipeline, we assessed how well the automatically generated protein families it produced matched expert-curated protein families from established databases that have undergone extensive curation, including multiple rounds of search and refinement (Figure 2). To do so, we collected protein sequences from 50 families (each containing at least 25 sequence members) from each of the following databases: NCBIFAM [34], PANTHER [35], Pfam [20], and HAMAP [36]. To explore diverse biological families and prevent overlap, each family was sampled from different branches of the InterPro hierarchy. The 200 families matched a total of 106,959 non-redundant sequences. To this sequence dataset, we added 10,000 additional sequences from UniProt-SwissProt and verified the absence of significant similarity to the pooled family proteins using *DIAMOND BLASTp* [37] with default parameters (>60%

sequence identity, the *tantan* repeat masking algorithm for alignments, and *e*-value ≤ 0.001). This resulted in a final input set of 116,959 unique protein sequences.

Subsequently, we executed the nf-core/proteinfamilies pipeline with the combined protein dataset as input, using the 'linclust' algorithm along with a clustering identity of 0.5, coverage of 0.9 (both strands, with the greedy set cover mode), and a minimum cluster size of 3 sequences, for the initial *seed* clusters generation. Using these parameters, the pipeline produced 5,826 *seed* clusters. The pipeline then recruited additional sequences into families via *hmmsearch*, capturing 97.72% of the original unique sequence identifiers (104,522 out of 106,959). Subsequently, families with a profile HMM match to another profile HMM spanning 100% of the length of the query HMM were eliminated to reduce redundancy, leaving 709 families. These 709 remaining families captured 96.66% of the original unique sequence identifiers (103,385 out of 106,959). Even with this redundancy-reduction step, a single input protein family can be represented by multiple families from the 709 families due to differences in length and match composition. Thus, the 103,385 sequences were represented by 178,279 sequence regions. Family sequence recall was highest for HAMAP (99.7% of sequences, 84,051 / 84,335), followed by Pfam (90.6%, 7,452 / 8,227), NCBIFAM (89.9%, 2,407 / 2,677), and PANTHER (80.8%, 9,475 / 11,720). 2,941 additional UniProt sequences were also grouped into 295 families (without any sequence from the original input families set) out of the 709 (41.61%), and 395 out of the 709 (55.71%) contained only sequences from the original families. Despite the DIAMOND BLASTp search, 19 families contained both original input family and UniProt-SwissProt sequences.

To better understand how well these newly generated families represent the original 200 curated families used as input, we calculated the Jaccard index for protein identifiers in the overlap between the generated and original families. 274 families out of the 414 new families containing input family sequences had a Jaccard index score of at least 0.5 (i.e., they contained at least 50% of the original family sequences), representing 173 distinct families out of the 200 original ones (Supplementary Table 1). Family representation across databases was highest for HAMAP (50/50), followed by NCBIFAM (44/50), Pfam (42/50), and PANTHER (37/50). 31 of the original families (15.5%) were represented by more than one new family, but differed in their sequence composition. An extreme example of such a case is the PANTHER family PTHR23500. This sugar transport protein family is a long transmembrane family (average sequence length: 499) containing 1,316 sequences. This input family was represented by 53 families, each with slightly different sequence lengths due to variability in the input *seed* alignments (but significantly overlapping, as they all pass the Jaccard score index). The number of produced families that would match original families for stricter Jaccard similarity score thresholds (i.e., > 0.5) can be seen in Supplementary Figure 1.

27 of the original 200 families were not represented among the 274 families. Eight families were highly divergent, such that MMseqs2 clustering could not produce seeds containing three or more sequences; hence, they were filtered out at that step. For example, NCBIFAM family TIGR01988 (IPR010971) had 31 original sequences, all of which were clustered as singletons, highlighting the diverse nature of the family. For another 16 families, while these were also divergent, initial *seed* alignments were produced, but were split into multiple small subfamilies such that the Jaccard similarity score with the original families was below 0.5 for each of them. For example, the PANTHER family PTHR23072 (IPR039527) was initially split into eight seed clusters with MMseqs2. After hmmsearch recruitment and family redundancy removal, two

families remained: use_case_1124 and use_case_427, matching 19 and 31 sequences from PTHR23072, respectively, with no overlap between them, leaving 40 sequences unmatched. The Pfam family PF09826 (IPR019198) contains 68 total beta propellers and was split into use_case_4917 (30 sequences, 550 average length) and use_case_893 (16 sequences, 534 average length), with six sequences in common between the two generated families, leaving 28 initial sequences unmatched. The PANTHER family PTHR21227 (IPR006676), with an average sequence length of 347 amino acids, was mapped by three generated families: use_case_1769, use_case_2952, and use_case_2953. Families use_case_1769 and use_case_2953 were similar in length (244 and 239, respectively) and shared 52 of their total 54 respective sequences, whereas use_case_2952 recruited 20 of the larger original sequences, leading to an average sequence length of 459. Finally, three original families (PF00459, PF09704, and PTHR33910) were each partially mapped by a single generated family, but with a Jaccard Score below 0.5. The generated family use_case_118 matched 21 proteins from PF00459, leaving 22 unmatched, and thus scored just below the set threshold of 0.5. Similarly, use_case_3397 matched 14 out of 37 proteins from PF09704, leaving 23 unmatched, and use_case_2154 matched 8 out of 28 proteins from PTHR33910, leaving 20 unmatched.

Focusing on those families that scored above the Jaccard index score threshold, 255 families out of the 274 matching families contained only original family sequences (Supplementary Table 2). The remaining 19 families (2.68% of the total 709) contained 27 sequences not found in the original families (note that these are different from the 19 that fell below the Jaccard score index). Concerned that the families were recruiting seemingly unrelated sequences, we investigated them in more detail to understand the nature of the matches (Supplementary Table 3). The family use_case_3613, based on NCBIFAM NF045717, matched three sequences from PTHR23500 (A0A3B6G0N7/23-139, A0A3B6U448/23-139, and A0A3B6ETY9/23-140) and three further sequences from UniProt-SwissProt (O25918/1-120, T2KMF4/1116-1235, and Q1QI44/18-124). NF045717 is a family of response regulator receiver domain proteins lacking a DNA-binding domain, but all additional matched sequences also contain the same response regulator domain. They have been excluded from this NCBIFAM presumably because the sequences in this family only have the Response Regulator domain, while the additional sequences contain additional accessory domains. The family use_case_2043, based on NCBIFAM TIGR03685, matched a sequence from PTHR20856 (A0A2G3AJI4/1-108) and another from UniProt-SwissProt (P10622/5-104). The TIGR03685 family contains large ribosomal subunit proteins from Archaea, and P10622 is also a large ribosomal subunit protein, explaining the recruitment. The PTHR20856 family contains longer sequences that encode DNA-directed RNA polymerase subunits involved in RNA synthesis. Nevertheless, the matched region of A0A2G3AJI4, residues 1-108, has a similar predicted structure to that of the TIGR03685 sequences; a disordered region followed by four short helices. The remaining 17 families contained either one or two sequences from the UniProt-SwissProt set. However, in all cases, the addition of these sequences to the family could be explained due to the presence of a common sequence motif. For example, use_case_1007 recruited all 2622 proteins from the HAMAP family of bacterial small ribosomal subunit proteins (uS14) (HAMAP accession: MF_01364_B), along with one additional UniProt-SwissProt sequence (A6MMU2). This sequence is also a small ribosomal subunit protein (uS14c) from the species *Illicium verum*, but neither the DIAMOND search nor the HAMAP entry identified this similarity.

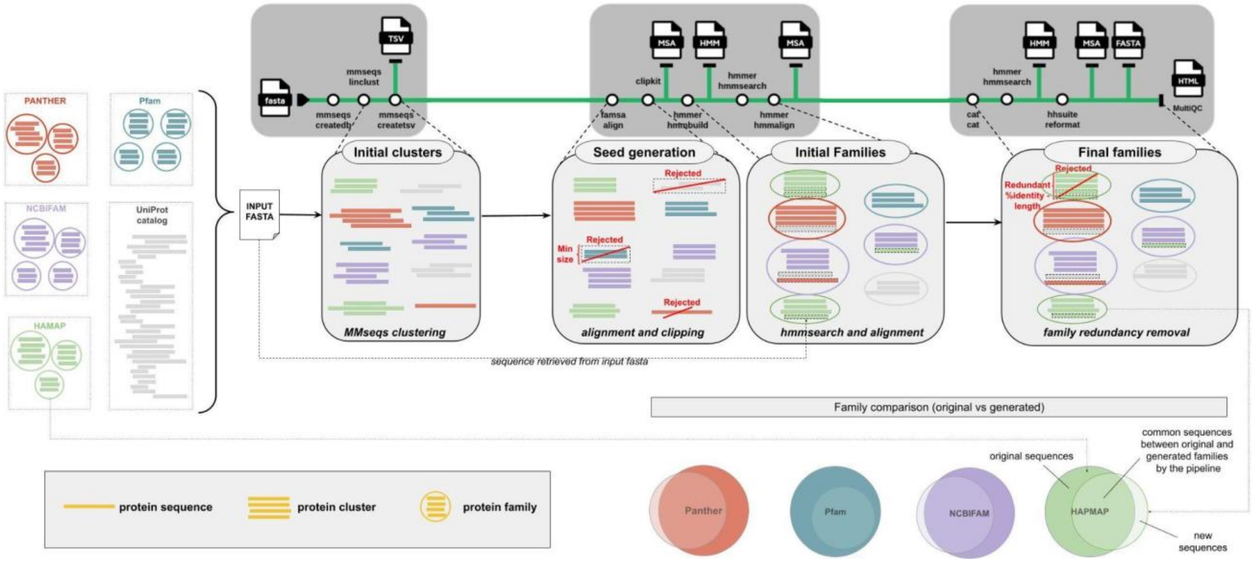

Figure 2. Workflow of the *nf-core/proteinfamilies* biological benchmark. First, 200 protein families are selected from diverse InterPro entries and merged with a set of seemingly unrelated UniProt-SwissProt sequences identified by a DIAMOND search. Next, the nf-core/proteinfamilies pipeline was applied to generate initial seed clusters, filtering out any cluster containing fewer than three sequences and creating an MSA (termed seed generation). Subsequently, the MSA is used to construct a profile HMM, which is then used to identify additional sequences related to the family. Finally, highly similar sequences are removed to reduce redundancy within the results. Overall, this process successfully incorporated 96.66% of the original family sequences into the output families, accurately reconstructing 173 of the 200 initial protein families.

We then carried out a series of additional reproducibility benchmarks to pinpoint the clustering and trimming parameter combinations that most affect the matched protein family results. We tried with the same parameters (cluster_seq_identity = 0.5, cluster_coverage = 0.9, cluster_cov_mode = 0, skip_msa_trimming = false) but with the *cluster* algorithm instead of *linclust*, since the number of sequences is of medium size and execution speed could be sacrificed for more sensitive initial clusters. This halved the total number of initial clusters, increasing their size, and finally matched five additional families (178 instead of 173) from NCBIFAM and PANTHER, due to fewer split families and initial sequences that did not cluster at all. Due to this increase in matched families, the *cluster* algorithm was used for all subsequent parameter combinations. All parameter combinations are listed in Supplementary Table 4, and the corresponding family-matching results are in Supplementary Table 5.

The most matched original families results were achieved by the `broad_and_trim` combination (cluster_seq_identity = 0.3, cluster_coverage = 0.5, cluster_cov_mode = 0, skip_msa_trimming = false), with a total of 192 matched out of the 200 original families, failing to reproduce only two Pfam and six PANTHER families. These eight unmatched families were split into multiple generated families. The same combination of parameters, but without alignment trimming, failed to produce 14 additional PANTHER families, indicating that some of the sampled PANTHER families contain one or more domains that are highly representative of the family. The results from using stricter identity and coverage combinations were even worse, mainly because they failed to produce enough initial clusters. This is somewhat expected because many of the protein families have been extensively iterated to represent a broader evolutionary range. Based

on these benchmark results, we tested a final parameter combination (the best-scoring) along with the greedy incremental MMseqs2 mode. This resulted in the same number of matched families, but with a slight increase in the number of initial clusters and final families, unnecessarily producing more groups and wasting more compute. Finally, by allowing the merging of generated families within our best-scoring `broad_and_trim` benchmark, with an hmmsearch length similarity score threshold of 0.9, we managed to generate 930 total families (97 fewer families than without merging) while also matching an additional (previously split) original family, matching a total of 193 out of the 200 initially sampled families.

Computational Benchmark

Following the protein family reproducibility benchmark, we then evaluated the computational performance of the *nf-core/proteinfamilies* workflow. To do so, the pipeline (version 1.0.0) was tested with UniRef90, a large-scale benchmark comprising 194,348,997 sequences (45GB compressed in FASTA format). The workflow execution was conducted on a SLURM High-Performance Computing (HPC) infrastructure, utilizing Singularity containers. The shared HPC cluster comprises 340 compute nodes, each equipped with 48 physical cores and 468 GB of RAM, and 20 memory nodes, each with 48 physical cores and 1977 GB of RAM.

One of the most resource-intensive stages of the pipeline is the initial MMseqs2 *linclust* clustering. A sequence identity of 50% and coverage of 90% for both strands were set as parameters. During this process, the pipeline utilized 150 GB of RAM. This step required the most CPUs (12) in the pipeline and took 93.5 minutes to complete. Upon completion, clusters with fewer than 100 members were filtered out, since the larger ones are more likely to yield high-quality *seed* alignments. This step produced 23,245 seed clusters. After inter-family redundancy removal (>80% model-length similarity), 4,921 families remained (~21% of the initial clusters). This reduction is expected, since we used a relaxed length threshold for model similarity (80%), which eliminated families that could have been sub-domains or super-families of other families, avoiding protein identifier duplication across the final set of families. Two histograms displaying the distribution of family sizes and representative sequence lengths are shown in Supplementary Figure 2.

Regarding the longest job durations, the FASTA partitioning process took approximately 3.3 hours, whereas the longest-running module was a FAMSA alignment process on an input containing over a million proteins, which required 33 hours to complete. On average, each FAMSA process took 4.7 hours, each *hmmsearch* process required 83 minutes, and extracting family representative sequences, which can be used for further downstream analyses (i.e., protein annotation, structural prediction), from Stockholm MSA files took 37 hours. Detailed resource statistics are available in Supplementary File 1.

Discussion

The ever-increasing size of protein databases limits the extent to which manual efforts for protein family curation can be applied. The open-source *nf-core/proteinfamilies* pipeline provides essential features that make it well-suited for automated, scalable protein family generation. It also ensures portability, modularity, reproducibility, transparency, and updating. The pipeline

design prioritizes interoperability, producing standardized outputs at every stage to allow smooth integration with other tools and workflows. The pipeline also leverages the caching mechanism of Nextflow, which allows interrupted runs to resume without recomputing completed steps. Hosted on GitHub by the nf-core organization, the pipeline can be readily executed on any machine using Nextflow and Java. Users can choose to run it with Conda environments *(conda-forge/bioconda)* [38] or using containerized modules via Docker [39] or Singularity [40], enabling seamless deployment across different operating systems and computational environments. With comprehensive documentation and support for both the command-line interface (CLI) and the nf-core graphical user interface (GUI), researchers of varying bioinformatics skill levels can navigate, configure, and use the pipeline effectively without extensive training.

The computational benchmarks (pipeline version 1.0.0) demonstrated that, despite handling vast datasets, the resource requirements remained moderate, but did require access to an HPC. The resource utilisation statistics also highlighted key bottlenecks in the pipeline, such as the time required to extract family representatives (for post-pipeline downstream analyses). After running this benchmark, we have addressed this performance issue by optimizing the module for extracting family representatives to enable parallel execution (as of v1.3.1). As this is a purely technical improvement that does not alter the output, we have not rerun our benchmarks with the updated pipeline. While the pipeline currently scales well, further optimizations such as improved parallel execution, the use of more efficient sequence-processing libraries, and dataset-size-specific resource allocation could further enhance computational performance, enabling continued scaling well into the future as sequence sets continue to grow.

As demonstrated by our protein family reproducibility benchmark, what constitutes a protein family differs according to the goal of the protein family database, for example, capturing isofunctional sequences versus broad capture of a functional fold or full-length sequences versus globular domains. Due to the nature of the initial seed generation and the strict sequence clustering coverage, our initial benchmark approach focused on matching conserved regions or domains rather than matching full-length proteins. Nevertheless, we were able to recreate a substantial set of protein families by placing 96.66% of sequences drawn from 200 InterPro families into 414 generated families, with only 19 of those recruiting additional UniProt-SwissProt sequences. A closer inspection of the 27 UniProt-SwissProt sequences matching the families revealed significant structure similarity and/or consistent annotations, indicating that these were not false-positive matches. With the advent of protein structure-based similarity searches sparked by the protein structure prediction revolution, these more distant relationships can now often be readily detected, overcoming this limitation.

27 of the original 200 families were not represented in the final set, as highly divergent families could not be generated using the benchmark's parameters, which is likely a limitation of the approach. Similarly, 31 original families were represented by more than one family, indicating that some of the curated families represented more divergent families, and that our unsupervised approach was unable to recapitulate them. Both types of families typically came from PANTHER or Pfam, which are recognised as representing more divergent families or domains. A series of follow-up benchmarks with different clustering and trimming parameter combinations highlighted that the approach to use when replicating protein families may differ, depending on the nature of the target protein family resource (e.g., domain-centric, full-length/multi-domain, and/or isofunctional). However, we managed to identify parameter values for which the nf-

core/proteinfamilies pipeline appears to replicate the original families at best across all four of the tested resources. This parameter combination consists of lower sequence-identity and coverage thresholds for clustering, along with trimming the gappy ends of the produced alignments, and managed to replicate 193 out of the 200 initially sampled families.

Certain sequence classes, such as transmembrane segments, coiled-coils, repeats, and intrinsically disordered regions are known to bias sequence seeding and profile building by generating short, low-complexity motifs. This behaviour has previously been documented and mitigated in search tools via masking and compositional corrections (e.g., MMseqs2; masking/TANTAN). As a result, the nf-core/proteinfamilies pipeline may produce fragmented families or leave such sequences unclustered, consistent with challenges observed in curated databases. While the current workflow does not include explicit handling of such cases, its modular design allows downstream integration with structure-based analyses and repeat/transmembrane/intrinsically disorder region annotation tools to further explore these effects.

While nf-core/proteinfamilies is focused on sequence-based clustering, structural similarity methods can provide a complementary means of assessing family purity. The increasing coverage of AlphaFoldDB and tools such as Foldseek [41] could be used to verify that family members share the same structural superfamily. The nf-core/proteinfold (https://github.com/nf-core/proteinfold) pipeline [42] can predict 3D structures for family representative sequences from nf-core/proteinfamilies and then identify their structural homologs using Foldseek. We enable this functionality by automatically generating a samplesheet for use as input in the downstream nf-core/proteinfold pipeline. After protein families are generated, applying annotations from curated databases is an obvious next step. To keep the scope modular, nf-core/proteinfamilies focuses exclusively on protein family generation, while annotation functionality is being developed in complementary pipelines such as nf-core/proteinannotator (https://github.com/nf-core/proteinannotator). We also enable the chaining of nf-core/proteinfamilies to nf-core/proteinannotator, by producing the respective downstream samplesheet with the family representative sequences. It is also important to note that predicting structures and annotating the millions of sequences generated by metagenomics studies at scale would incur substantial computational cost. By running the nf-core/proteinfamilies pipeline in between, we enable the reduction of the search space for downstream analyses by highlighting a representative sequence per family, as structural features and functional annotations are expected to be largely transferable among family members.

The automatic generation of protein families at scale enables the exploration of vast sequence collections and provides starting points for manual curation. Redundancy removal mechanisms can reduce storage requirements while retaining both family and sequence diversity. The family updating mechanism enables updating of alignments and models when new sequences become available, without the need to recluster the entire sequence database, and allows persistent identifiers to be established. With its robustness, scalability, portability, and ease of use, we anticipate that the *nf-core/proteinfamilies* pipeline will be widely adopted for protein family generation across various bioinformatics applications, further advancing research in the field.

Availability

The *nf-core/proteinfamilies* pipeline is implemented using Nextflow DSL2 and follows nf-core pipeline standards. The source code is available on GitHub at https://github.com/nf-core/proteinfamilies, and each release is archived on Zenodo (current latest release: 2.1.0, DOI: 10.5281/zenodo.15373136). For HPC infrastructures, the pipeline supports one of the available centralized nf-core configurations (currently more than 150): https://github.com/nf-core/configs. Detailed documentation is available at https://nf-co.re/proteinfamilies, and the pipeline can be executed via the CLI or the nf-core GUI on the same website. The computational benchmark results (MSA and HMM files) are available at: https://zenodo.org/records/14997773. The reproducibility benchmark original family FASTA files, additional UniProt-SwissProt sequences, and generated family models and alignments can be found at: https://zenodo.org/records/16634647. The pipeline has also been deposited to SciCrunch (RRID: SCR_027374), bio.tools (https://bio.tools/nf-core_proteinfamilies), and workflowhub.eu (DOI: 10.48546/workflowhub.workflow.1954.3).

Funding

E.K. was funded from the European Union's Horizon 2020 research and innovation program under the Marie Skłodowska-Curie grant agreement No 945405; R.F., M.B.C., and L.R. were funded by EMBL; G.A.P. was supported by: Hellenic Foundation for Research and Innovation (H.F.R.I.) under the 'Third Call for H.F.R.I. Research Projects to support faculty members and researchers' [23592 - EMISSION]; F.A.B. was supported by: Hellenic Foundation for Research and Innovation (H.F.R.I.) under the '4th Call for H.F.R.I. Research Project to support Postdoctoral Researchers' [28787-VIROMINE]; I.G.S. was supported by startup funds from the Penn State College of Medicine and the University of Texas at Austin; The work conducted by N.C.K. in the US Department of Energy Joint Genome Institute (https://ror.org/04xm1d337) was supported by the US Department of Energy Office of Science user facilities, operated under contract no. DE-AC02-05CH11231

References

1. Aplakidou E, Vergoulidis N, Chasapi M, Venetsianou NK, Kokoli M, Panagiotopoulou E, et al.. Visualizing metagenomic and metatranscriptomic data: A comprehensive review. *Comput Struct Biotechnol J*. 2024; doi: 10.1016/j.csbj.2024.04.060.
2. Baltoumas FA, Karatzas E, Paez-Espino D, Venetsianou NK, Aplakidou E, Oulas A, et al.. Exploring microbial functional biodiversity at the protein family level-From metagenomic sequence reads to annotated protein clusters. *Front Bioinform*. 2023; doi: 10.3389/fbinf.2023.1157956.
3. The UniProt Consortium, Bateman A, Martin M-J, Orchard S, Magrane M, Ahmad S, et al.. UniProt: the Universal Protein Knowledgebase in 2023. *Nucleic Acids Research*. 2023; doi: 10.1093/nar/gkac1052.
4. Berman HM. The Protein Data Bank. *Nucleic Acids Research*. 2000; doi: 10.1093/nar/28.1.235.
5. O'Leary NA, Wright MW, Brister JR, Ciufo S, Haddad D, McVeigh R, et al.. Reference sequence (RefSeq) database at NCBI: current status, taxonomic expansion, and

functional annotation. *Nucleic Acids Res*. 2016; doi: 10.1093/nar/gkv1189.
6. Sayers EW, Cavanaugh M, Clark K, Pruitt KD, Schoch CL, Sherry ST, et al.. GenBank. *Nucleic Acids Research*. 2022; doi: 10.1093/nar/gkab1135.
7. Chen I-MA, Chu K, Palaniappan K, Ratner A, Huang J, Huntemann M, et al.. The IMG/M data management and analysis system v.7: content updates and new features. *Nucleic Acids Research*. 2023; doi: 10.1093/nar/gkac976.
8. Jumper J, Evans R, Pritzel A, Green T, Figurnov M, Ronneberger O, et al.. Highly accurate protein structure prediction with AlphaFold. *Nature*. 2021; doi: 10.1038/s41586-021-03819-2.
9. Richardson L, Allen B, Baldi G, Beracochea M, Bileschi ML, Burdett T, et al.. MGnify: the microbiome sequence data analysis resource in 2023. *Nucleic Acids Research*. 2023; doi: 10.1093/nar/gkac1080.
10. Mistry J, Chuguransky S, Williams L, Qureshi M, Salazar GA, Sonnhammer ELL, et al.. Pfam: The protein families database in 2021. *Nucleic Acids Res*. 2021; doi: 10.1093/nar/gkaa913.
11. Paysan-Lafosse T, Blum M, Chuguransky S, Grego T, Pinto BL, Salazar GA, et al.. InterPro in 2022. *Nucleic Acids Res*. 2023; doi: 10.1093/nar/gkac993.
12. Baltoumas FA, Karatzas E, Liu S, Ovchinnikov S, Sofianatos Y, Chen I-M, et al.. NMPFamsDB: a database of novel protein families from microbial metagenomes and metatranscriptomes. *Nucleic Acids Res*. 2024; doi: 10.1093/nar/gkad800.
13. Pavlopoulos GA, Baltoumas FA, Liu S, Selvitopi O, Camargo AP, Nayfach S, et al.. Unraveling the functional dark matter through global metagenomics. *Nature*. 2023; doi: 10.1038/s41586-023-06583-7.
14. Sillitoe I, Cuff AL, Dessailly BH, Dawson NL, Furnham N, Lee D, et al.. New functional families (FunFams) in CATH to improve the mapping of conserved functional sites to 3D structures. *Nucleic Acids Research*. Oxford University Press (OUP); 2012; doi: 10.1093/nar/gks1211.
15. Huerta-Cepas J, Szklarczyk D, Heller D, Hernández-Plaza A, Forslund SK, Cook H, et al.. eggNOG 5.0: a hierarchical, functionally and phylogenetically annotated orthology resource based on 5090 organisms and 2502 viruses. *Nucleic Acids Research*. 2019; doi: 10.1093/nar/gky1085.
16. Cantalapiedra CP, Hernández-Plaza A, Letunic I, Bork P, Huerta-Cepas J. eggNOG-mapper v2: Functional Annotation, Orthology Assignments, and Domain Prediction at the Metagenomic Scale. Tamura K, editor. *Molecular Biology and Evolution*. 2021; doi: 10.1093/molbev/msab293.
17. Kanehisa M, Sato Y, Kawashima M, Furumichi M, Tanabe M. KEGG as a reference resource for gene and protein annotation. *Nucleic Acids Res*. 2016; doi: 10.1093/nar/gkv1070.
18. Galperin MY, Vera Alvarez R, Karamycheva S, Makarova KS, Wolf YI, Landsman D, et al.. COG database update 2024. *Nucleic Acids Research*. 2024; doi: 10.1093/nar/gkae983.
19. Tatusov RL, Fedorova ND, Jackson JD, Jacobs AR, Kiryutin B, Koonin EV, et al.. The COG database: an updated version includes eukaryotes. *BMC Bioinformatics*. 2003; doi: 10.1186/1471-2105-4-41.
20. Paysan-Lafosse T, Andreeva A, Blum M, Chuguransky SR, Grego T, Pinto BL, et al.. The Pfam protein families database: embracing AI/ML. *Nucleic Acids Res*. 2025; doi: 10.1093/nar/gkae997.
21. Steinegger M, Söding J. MMseqs2 enables sensitive protein sequence searching for the analysis of massive data sets. *Nat Biotechnol*. 2017; doi: 10.1038/nbt.3988.
22. Fu L, Niu B, Zhu Z, Wu S, Li W. CD-HIT: accelerated for clustering the next-generation sequencing data. *Bioinformatics*. Oxford University Press (OUP); 2012; doi: 10.1093/bioinformatics/bts565.

23. Di Tommaso P, Chatzou M, Floden EW, Barja PP, Palumbo E, Notredame C. Nextflow enables reproducible computational workflows. *Nat Biotechnol*. 2017; doi: 10.1038/nbt.3820.
24. Ewels PA, Peltzer A, Fillinger S, Patel H, Alneberg J, Wilm A, et al.. The nf-core framework for community-curated bioinformatics pipelines. *Nat Biotechnol*. 2020; doi: 10.1038/s41587-020-0439-x.
25. Langer BE, Amaral A, Baudement M-O, Bonath F, Charles M, Chitneedi PK, et al.. Empowering bioinformatics communities with Nextflow and nf-core.
26. Telatin A, Fariselli P, Birolo G. SeqFu: A Suite of Utilities for the Robust and Reproducible Manipulation of Sequence Files. *Bioengineering*. 2021; doi: 10.3390/bioengineering8050059.
27. Shen W, Sipos B, Zhao L. SeqKit2: A Swiss army knife for sequence and alignment processing. *iMeta*. Wiley; 2024; doi: 10.1002/imt2.191.
28. Deorowicz S, Debudaj-Grabysz A, Gudyś A. FAMSA: Fast and accurate multiple sequence alignment of huge protein families. *Sci Rep*. 2016; doi: 10.1038/srep33964.
29. Katoh K, Standley DM. MAFFT multiple sequence alignment software version 7: improvements in performance and usability. *Mol Biol Evol*. 2013; doi: 10.1093/molbev/mst010.
30. Steenwyk JL, Buida TJ, Li Y, Shen X-X, Rokas A. ClipKIT: A multiple sequence alignment trimming software for accurate phylogenomic inference. *PLoS Biol*. 2020; doi: 10.1371/journal.pbio.3001007.
31. Finn RD, Clements J, Eddy SR. HMMER web server: interactive sequence similarity searching. *Nucleic Acids Res*. 2011; doi: 10.1093/nar/gkr367.
32. Ewels P, Magnusson M, Lundin S, Käller M. MultiQC: summarize analysis results for multiple tools and samples in a single report. *Bioinformatics*. 2016; doi: 10.1093/bioinformatics/btw354.
33. Santus L, Garriga E, Deorowicz S, Gudyś A, Notredame C. Towards the accurate alignment of over a million protein sequences: Current state of the art. *Curr Opin Struct Biol*. 2023; doi: 10.1016/j.sbi.2023.102577.
34. Li W, O'Neill KR, Haft DH, DiCuccio M, Chetvernin V, Badretdin A, et al.. RefSeq: expanding the Prokaryotic Genome Annotation Pipeline reach with protein family model curation. *Nucleic Acids Res*. 2021; doi: 10.1093/nar/gkaa1105.
35. Thomas PD, Kejariwal A, Campbell MJ, Mi H, Diemer K, Guo N, et al.. PANTHER: a browsable database of gene products organized by biological function, using curated protein family and subfamily classification. *Nucleic Acids Res*. 2003; doi: 10.1093/nar/gkg115.
36. Lima T, Auchincloss AH, Coudert E, Keller G, Michoud K, Rivoire C, et al.. HAMAP: a database of completely sequenced microbial proteome sets and manually curated microbial protein families in UniProtKB/Swiss-Prot. *Nucleic Acids Res*. 2009; doi: 10.1093/nar/gkn661.
37. Buchfink B, Xie C, Huson DH. Fast and sensitive protein alignment using DIAMOND. *Nat Methods*. 2015; doi: 10.1038/nmeth.3176.
38. Grüning B, Dale R, Sjödin A, Chapman BA, Rowe J, Tomkins-Tinch CH, et al.. Bioconda: sustainable and comprehensive software distribution for the life sciences. *Nat Methods*. 2018; doi: 10.1038/s41592-018-0046-7.
39. Merkel D. Docker: lightweight Linux containers for consistent development and deployment. *Linux J*. 2014:2:22014;
40. Kurtzer GM, Sochat V, Bauer MW. Singularity: Scientific containers for mobility of compute. *PLoS One*. 2017; doi: 10.1371/journal.pone.0177459.
41. Van Kempen M, Kim SS, Tumescheit C, Mirdita M, Lee J, Gilchrist CLM, et al.. Fast and accurate protein structure search with Foldseek. *Nat Biotechnol*. 2024; doi:

10.1038/s41587-023-01773-0.
42. Baltzis A, Espinosa-Carrasco J, Santus L, Mansouri L, Steinegger M, Patel H, et al.. nf-core/proteinfold: a bioinformatics best-practice analysis pipeline for protein 3D structure prediction. Zenodo;

Figure 1

Click here to access/download;Figure;proteinfamilies_workflow_light.png

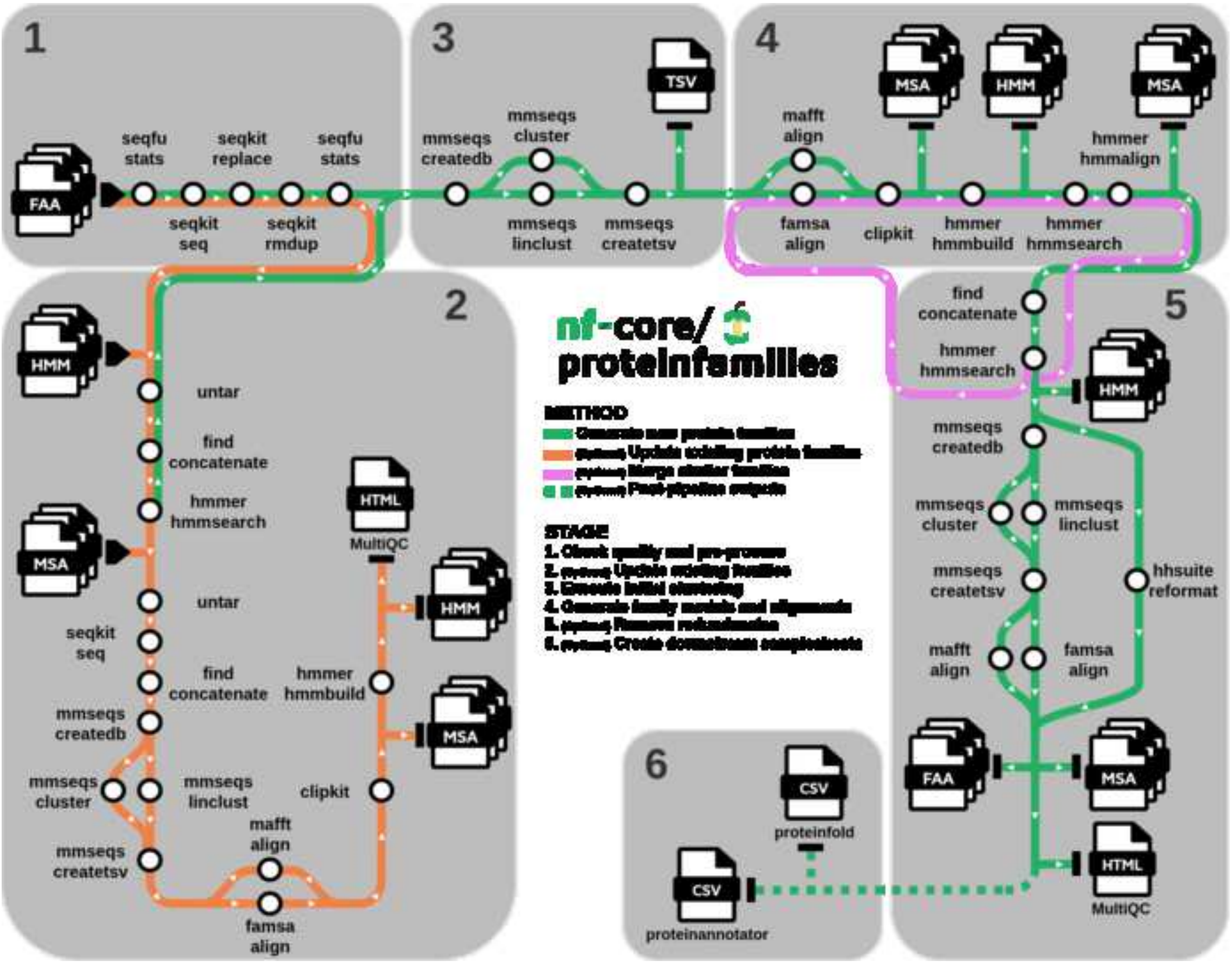

Figure 2 Click here to access/download;Figure;protein_families_benchmark.png

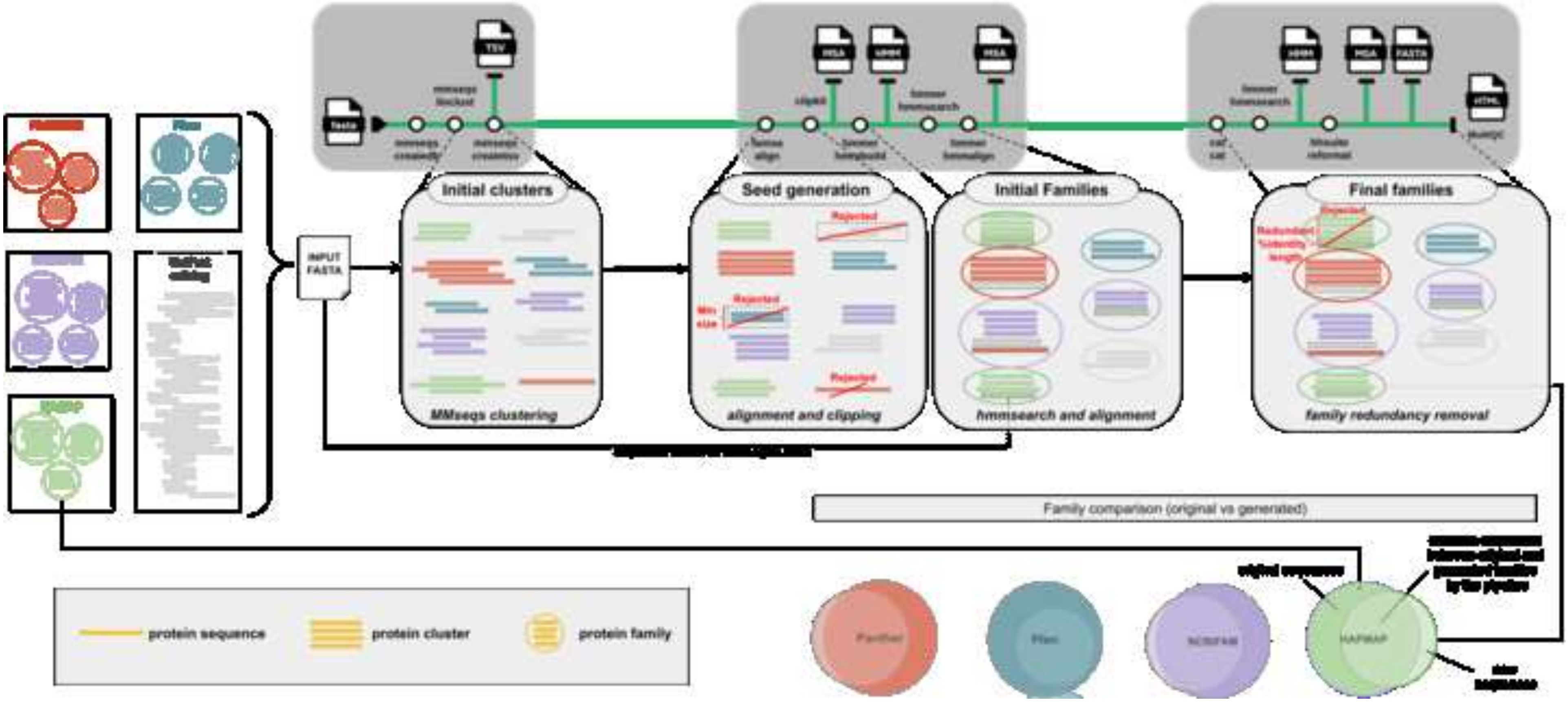

Supplementary File 1

Click here to access/download
Supplementary Material
Supplementary File 1.pdf

Supplementary Figure 1

Click here to access/download
Supplementary Material
Supplementary Figure 1.png

Supplementary Figure 2

Click here to access/download
Supplementary Material
Supplementary Figure 2.png

Supplementary Table 1

Click here to access/download
Supplementary Material
Supplementary Table 1.xlsx

Supplementary Table 2

Click here to access/download
Supplementary Material
Supplementary Table 2.xlsx

Supplementary Table 3

Click here to access/download
Supplementary Material
Supplementary Table 3.xlsx

Supplementary Table 4

Click here to access/download
Supplementary Material
Supplementary Table 4.xlsx

Supplementary Table 5

Click here to access/download
Supplementary Material
Supplementary Table 5.xlsx
