# Supplementary material for: nf-core/proteinfamilies: a scalable pipeline for the generation of protein families
Source: Gigascience. 2026 Jan 21;15:giag009. doi: 10.1093/gigascience/giag009 (PMC12950615; doi:10.1093/gigascience/giag009)
Supplement: giag009_Authors_Response_To_Reviewer_Comments_original_submission [file giag009_authors_response_to_reviewer_comments_original_submission.pdf]

Author's Response To Reviewer Comments

We thank the editor and reviewers for the comments that helped us upgrade the quality of the pipeline and the manuscript. The main revision updates can be seen in the manuscript in red color. For further FAIR compliance, the following pipeline identifiers have been registered;
SciCrunch, RRID:SCR_027374 https://rrid.site/data/record/nlx_144509-1/RRID:SCR_027374/resolver?q=SCR_027374&i=rrid:scr_027374
workflowhub.eu https://workflowhub.eu/workflows/1954 latest version DOI: 10.48546/workflowhub.workflow.1954.3
bio.tools: https://bio.tools/nf-core_proteinfamilies

A point-by-point response to reviewer comments follows:

Reviewer #1
The authors present nf-core/proteinfamilies, a standardized Nextflow workflow that constructs protein families de novo or classifies sequences against existing families. Using a curated 200-family benchmark and a UniRef90-scale run, the authors show that the pipeline attains high recall with efficient runtimes. Given the ever-increasing size of sequence databases, this work is timely and fills a practical gap in reproducible, at-scale family curation; I expect it to be adopted widely by many research groups.

I have several comments and suggestions below:

1. In my view, this workflow will, by construction, yield a mixture of families: some anchored on a single conserved domain/segment, others centered on recurrent multi-domain cores, and some that capture the full-length sequence. This differs from widely used family databases: Pfam is largely domain-level, whereas HAMAP and NCBIFAM are mostly full-length/isofunctional (with PANTHER sitting in between). The resulting granularity is largely determined by MMseqs2 settings (sequence identity, query/target coverage, coverage mode) and by any alignment trimming, which biases toward conserved cores. Please add a brief discussion making this explicit, with practical guidance for tuning toward full-length versus domain-centric generation of families.

Reply:
We thank the reviewer for this comment. We agree that nf-core/proteinfamilies can produce a spectrum of family types (as found in Pfam), ranging from single domain families, to families containing multiple domains to full-length protein families. We have revised the manuscript to highlight the main parameters that can influence this granularity, offering practical guidance on how users can tune them to bias clustering toward domain-centric or full-length families, depending on the intended use case.

The “Parametrization” paragraph now reads:
Setting higher MMseqs2 coverage thresholds (e.g., ≥0.8) with alignment trimming enabled tends to bias protein family results toward conserved domains, whereas lower coverage thresholds (e.g., ≤0.5) with skipped trimming tend to produce more full-length or multi-domain families.

We also rephrased the relevant sentence in the discussion, which now reads:
Due to the nature of the initial seed generation and the strict sequence clustering coverage, our initial benchmark approach focused on matching conserved regions or domains rather than matching full-length proteins.

I also recommend a parameter-sensitivity analysis on the 200-family set: sequence identity (30-70%), coverage thresholds (50-95%), and coverage mode (query/target/both), with and without trimming. For each setting, report (i) total families and split/merge rates per curated family, and (ii) a simple granularity readout, the proportion classified as domain-anchored, multi-domain, or full-length. This would clarify how parameter choices drive family counts and domain/full-length centricity, and help readers select defaults aligned with their use case.

Reply:
This comment was very useful for setting the default clustering parameters in the nf-core/proteinfamilies pipeline configuration, and we thank the reviewer for this. Based on the suggested parameter options, we conducted a series of benchmarks using the same 200 original families described in the reproducibility paragraph of the paper.

The original benchmark was run with the linclust algorithm and with the following parameters:
cluster_seq_identity = 0.5, cluster_coverage = 0.9, cluster_cov_mode = 0, trim_msa = true
The cluster_cov_mode = 0 indicates that both strands were checked for coverage and that MMseqs2 is using the greedy set-cover clustering mode.

For the new combinations of suggested benchmarks, we first tried the same parameters with the cluster algorithm, since the number of sequences is medium, and execution speed can be sacrificed for more sensitive initial clusters. This halved the total number of initial clusters, increasing their size, and finally matched five additional families (178 instead of 173) from NCBIFAM and PANTHER due to fewer split families and initial sequences that did not cluster at all. With this in mind, we decided to continue the remaining benchmark combinations with the cluster algorithm.

All parameter combinations are listed in the new Supplementary Table 4, and the corresponding family-matching results are in Supplementary Table 5. The best results were achieved by the `broad_and_trim` combination (cluster_seq_identity = 0.3, cluster_coverage = 0.5, cluster_cov_mode = 0, trim_msa = true), with a total of 192/200 matched families, only failing to match 2 Pfam and 6 PANTHER families, which did not disappear, but were split into more than one of the generated families. The same configuration but without trimming, `lost` 14 additional PANTHER families, indicating that the sampled PANTHER families are probably domain-centric. The stricter coverage combinations lagged, mainly because they failed to produce enough initial clusters. This is somewhat expected because many of the protein families have been extensively iterated to represent a broader evolutionary range. Based on these benchmarks, we also tried another MMseqs2 coverage and clustering mode (greedy incremental) along with the best-scoring parameters. This resulted in the same number of matched families; however, it led to a small increase in the number of initial clusters and final families, thereby unnecessarily producing more groups and wasting more compute.

The reproducibility benchmark and discussion paragraphs have been updated accordingly.

2. In the results, the splits/misses are concentrated in Pfam/PANTHER, while HAMAP/NCBIFAM are much closer to one-to-one (HAMAP 50/50). This suggests the inflated family count is driven, in part, by the domain-centric portion of the benchmark rather than the method itself. Please add a brief note in the Discussion to make this explicit.

Reply:
We thank the reviewer for this comment. According to the additional benchmarks we ran, HAMAP was the easiest to reproduce across all experiments. Following, NCBIFam was also replicated the most, except when the identity and coverage parameters became too strict (0.7 and 0.95, respectively), in which case only 29 of its 50 original families were matched. PANTHER and Pfam matched percentages were indeed more related to the clustering parameters. Both were best replicated when clustering thresholds were more relaxed. Only PANTHER was affected by the alignment trimming, and only during the broader clustering experiment; not trimming the alignments affected the results, matching 14 fewer original families. This indicates that the approach to use when replicating protein families may differ depending on the nature of the target protein family resource (domain-centric, full-length, isofunctional, etc.).

However, through reproducibility benchmarks, we identified default parameter values for which the nf-core/proteinfamilies pipeline appears to replicate the original families at best across all four tested resources. The Discussion paragraph has been updated accordingly and now reads: “A series of follow-up benchmarks with different clustering and trimming parameter combinations highlighted that the approach to use when replicating protein families may differ, depending on the nature of the target protein family resource (e.g., domain-centric, full-length/multi-domain, and/or isofunctional). However, we managed to

identify parameter values for which the nf-core/proteinfamilies pipeline appears to replicate the original families at best across all four of the tested resources. This parameter combination consists of lower sequence-identity and coverage thresholds for clustering, along with trimming the gappy ends of the produced alignments, and managed to replicate 193 out of the 200 initially sampled families."

3. Since AFDB has models for most UniProt entries, could these models be used as an orthogonal purity check of the generated families, e.g., map members to AFDB and ask whether they cluster to the same fold by TM-score/Foldseek (allowing full-length differences when the family is domain-anchored)?

Reply:
We thank the reviewer for this suggestion. We agree that AlphaFold DB models, combined with structural comparison tools such as Foldseek, provide a promising, orthogonal strategy for assessing family purity at the fold level. However, nf-core/proteinfamilies is designed as a sequence-based family-generation workflow and does not currently include structure-prediction or structure-clustering modules. These tasks are the focus of complementary pipelines within the nf-core community (e.g., nf-core/proteinfold), and our long-term goal is to support the chaining of these complementary pipelines. In fact, we implemented the required pipeline chaining (i.e., nf-core/proteinfamilies to nf-core/proteinfold) that automatically prepares a downstream input samplesheet for nf-core/proteinfold in the latest release version of nf-core/proteinfamilies (2.1.0). This pipeline chaining functionality enables seamless structure prediction of the generated family representative sequences and structural annotation with Foldseek, matching homologs from any desired structure database (e.g., AlphaFoldDB, PDB). We have added a note in the Discussion to clarify this scope. The paragraph now reads: "While nf-core/proteinfamilies is focused on sequence-based clustering, structural similarity methods can provide a complementary means of assessing family purity. The increasing coverage of AlphaFoldDB and tools such as Foldseek could be used to verify that family members share the same structural superfamily. The nf-core/proteinfold (https://github.com/nf-core/proteinfold) pipeline can predict 3D structures for family representative sequences from nf-core/proteinfamilies and then identify their structural homologs using Foldseek. We enable this functionality by automatically generating a samplesheet for use as input in the downstream nf-core/proteinfold pipeline"

4. HHsearch-based merging of divergent splits. In my view, and the authors note this, several curated families split simply because sequences are very divergent. An optional HHsearch (HMM-HMM) pass could merge these back: merge only at high probability (≈≥95%) with reciprocal coverage of the shorter model (≥0.6). It would be useful to include this as an optional stage in the pipeline.

Reply:
We thank the reviewer for the comment. This turned out to be a very valuable addition to the pipeline. We decided to create a new subworkflow (i.e., merge_families) that would sit between the family redundancy and the sequence redundancy removal. It makes sense, as this step reduces code duplication by either filtering out redundant, or merging similar families, before proceeding to sequence removal and full MSA rebuilding. However, instead of using additional new modules from the HHsuite library, we decided to reuse the results of the existing hmmsearch module that was used to identify family-level redundancies (i.e., family reps against family models). We now provide a second, lower-length threshold parameter in the IDENTIFY_REDUNDANT_FAMS module, which also outputs the similarity scores between family representative sequences and family profile HMMs. Above a user-defined similarity threshold (e.g., 0.9, – should be below the redundancy threshold, which was 1 for our benchmarks), the seed alignments of these families may be merged. The family-generation subworkflow will then be re-triggered for these merged clusters (align/trim/build hmm/recruit sequences/generate full alignment). Testing this new subworkflow against our benchmarks, with a similarity score of 0.9, we generated 930 families (97 fewer than the best parameter combination –broad_and_trim) while also matching an additional (previously split) original family. We carried out more experiments with lower length similarity thresholds, but then the benchmark would start failing to match all 50 HAMAP families.

The "Methodology/Redundancy removal" paragraph has been updated to read:
"Achieving a lower matching length threshold (e.g., 90%) still indicates similarity between families. The pipeline can optionally merge the seed alignments for such families and proceed to generate updated profile HMMs and full alignments."

The “Protein Family Reproducibility Benchmark” paragraph has also been updated to read:
“Finally, by allowing the merging of generated families within our best-scoring `broad_and_trim` benchmark, with an hmmsearch length similarity score threshold of 0.9, we managed to generate 930 total families (97 fewer families than without merging) while also matching an additional (previously split) original family, matching a total of 193 out of the 200 initially sampled families.”

5. Optional annotation of de novo families. I think it would be useful to add an annotation step that compares each de novo family (family HMM or MSA) against curated resources (Pfam, NCBIFAM, PANTHER/HAMAP).

Reply:
We thank the reviewer for this suggestion. Annotation of protein families against curated resources (Pfam, NCBIFAM, PANTHER, HAMAP, FunFams, etc.) is indeed an essential step in downstream analysis. However, we have intentionally scoped the nf-core/proteinfamilies pipeline to focus only on de novo family generation and updating. Incorporating annotation directly here would duplicate functionality that is under active development in other nf-core pipelines (e.g., nf-core/proteinannotator). Instead, we support chaining these workflows so that users can run annotation as a dedicated stage after family generation, for example, on the output fasta file containing all family representative sequences. We recently added the hmmsearch module in the nf-core/proteinannotator pipeline to annotate sequence domains with the latest Pfam release (https://github.com/nf-core/proteinannotator/pull/61) as well as with functional domains from FunFams (https://github.com/nf-core/proteinannotator/pull/62). We aim to expand the domain annotation databases to optionally include NCBIFAM, PANTHER, and HAMAP in the future. We have added a note in the Discussion to clarify this design decision and to highlight that nf-core/proteinfamilies outputs (MSAs, HMMs, representative sequences) are structured precisely to enable seamless integration with downstream analysis.

The paragraph now reads: “After protein families are generated, applying annotations from curated databases is an obvious next step. To keep the scope modular, nf-core/proteinfamilies focuses exclusively on protein family generation, while annotation functionality is being developed in complementary pipelines such as nf-core/proteinannotator (https://github.com/nf-core/proteinannotator). We also enable the chaining of nf-core/proteinfamilies to nf-core/proteinannotator, by producing the respective downstream samplesheet with the family representative sequences.”

Immediately after this we also added two sentences, describing the cost benefits of running the nf-core/proteinfamilies before heavy-compute downstream analyses such as structural prediction and the application of functional annotations:
“It is also important to note that predicting structures and annotating the millions of sequences generated by metagenomics studies at scale would incur substantial computational cost. By running the nf-core/proteinfamilies pipeline in between, we enable the reduction of the search space for downstream analyses by highlighting a representative sequence per family, as structural features and functional annotations are expected to be largely transferable among family members.”

6. Could you briefly outline your expectations for how the pipeline handles transmembrane segments, coiled-coils, repeats, and IDRs, classes prone to over-splitting under MMseqs2 seeding and trimming due to short-motif signal, low complexity, and variable lengths?

Reply:
We thank the reviewer for raising this question. Certain sequence classes (transmembrane segments, coiled-coils, repeats, and intrinsically disordered regions) are known to generate short, low-complexity motif signals that bias profile building, often leading to overly sensitive models and large clusters. This issue is recognized in the design of fast search tools (for example, MMseqs2 uses TANTAN masking to mitigate such effects), and the ability to generate seed clusters for such edge cases is typically part of the tool's parametrization. An example benchmark output of the pipeline is the sampled original family PF02996 –a chaperone protein containing six coiled coils. Even though the proteins from this family could not cluster (<3 members) with the parameter combination of cluster_seq_identity = 0.5, cluster_coverage

= 0.9, both for linclust and cluster algorithms (i.e., original_benchmarks –Supplementary Table 4–), tuning these parameters to 0.3 and 0.7, respectively (i.e., broad_and_trim), allowed the replication of this Prefoldin alpha-like protein family. Further use cases include the PF01032 and PF03169 families of transmembrane transporters, which also failed to cluster during the original_benchmarks. However, during the broad_and_trim benchmark, PF01032 was matched to a generated family, whereas the members of PF03169 were split across four generated families, and four PF03169 sequences were completely left out of the seed clusters. The rest of the seven non-matched families (i.e., split) include another transmembrane transporter (PTHR43653), three enzyme families (PTHR10030, PTHR21227, PTHR48077), one transferase (PTHR23072), one Translation initiation factor (PTHR43381), and one CRISPR-Cas (PF09704) protein family.

Added a paragraph in Discussion
"Certain sequence classes, such as transmembrane segments, coiled-coils, repeats, and intrinsically disordered regions are known to bias sequence seeding and profile building by generating short, low-complexity motifs. This behaviour has previously been documented and mitigated in search tools via masking and compositional corrections (e.g., MMseqs2; masking/TANTAN). As a result, the nf-core/proteinfamilies pipeline may produce fragmented families or leave such sequences unclustered, consistent with challenges observed in curated databases. While the current workflow does not include explicit handling of such cases, its modular design allows downstream integration with structure-based analyses and repeat/transmembrane/intrinsically disorder region annotation tools to further explore these effects."

Reviewer #2
This manuscript presents a Nextflow pipeline (nf-core/proteinfamilies) for large-scale protein-family generation. Overall, I think the paper is well written and clear. The pipeline appears very useful, and the reported results show good performance in both family reproducibility and computational efficiency.

I have a few minor comments requesting additional details:

1) Does the quality-check step only compute statistics, or is it also used to filter/clean the input set? If so, please specify the criteria and whether filtered sequences are excluded downstream.

Reply:
We thank the reviewer for this comment. This comment inspired us to update the quality check (QC) subworkflow, which previously only generated statistics, to now also enable sequence preprocessing, so users do not have to do it manually/externally before running the nf-core/proteinfamilies pipeline. From pipeline release version 1.4.0 and beyond, the QC subworkflow now produces statistics of the input sequences, re-formats (i.e., to upper case, removing gaps) and validates them, filters by user-defined min and max length thresholds (defaults [30, 5000]), removes duplicate sequences by name, and finally produces another QC statistics report after pre-processing. The SeqFu library is used to produce the statistics and MultiQC web reports, while the SeqKit library is used for the pre-processing steps. The "Quality check and pre-processing" paragraph of the "Methodology" section have been updated accordingly.

2) Which MMseqs2 clustering mode is used (set cover, connected components, or greedy)? Can this be changed within the pipeline? If configurable, please indicate the relevant parameters.

Reply:
To clarify the point concerning configurability, we have added two sentences in the beginning of the parametrization paragraph, stating:

"Pipeline parameters can be directly specified in the nextflow.config file, or be overridden by either providing an additional configuration file or by directly setting them in the run command. Furthermore, any tool parameters not exposed as Nextflow variables can be set via the ext.args variable for each process in the modules.config file."

Addressing the comment about the clustering mode, the nf-core/proteinfamilies pipeline uses MMseqs2 for initial sequence clustering. It exposes all relevant MMseqs2 parameters to the user, allowing the clustering mode to be set as desired. As the Reviewer indicates, in MMseqs2, the clustering mode can be set either directly via the --cluster-mode parameter or indirectly via the --cov-mode parameter, which automatically selects the most appropriate clustering mode for each case (which can also be overridden by the user). In the nf-core/proteinfamilies modules configuration file, we have set the --cov-mode argument to read the configurable cluster_cov_mode parameter (default value = 0) from the Nextflow configuration file, for both MMseqs2 algorithms (i.e., cluster, linclust). This implies a bidirectional sequence coverage, and the clustering mode is automatically set to the greedy set cover mode. To clarify this in the manuscript, we added the following sentences in the first paragraph of the parametrization section:

"The default bidirectional value for coverage mode (cluster_cov_mode = 0) automatically sets the MMseqs2 clustering to greedy set cover mode. However, users can override this parameter either indirectly, by changing the coverage mode, or directly, by setting the --cluster-mode argument in the modules configuration file."

3) In the reproducibility benchmark, you use DIAMOND BLASTp to assess similarity between the initial sequence set for the selected families and additional Swiss-Prot sequences. Which sequence identity and alignment coverage (if any) thresholds were applied?

Reply:
We thank the reviewer for the comment. The default values of DIAMOND BLASTp have now been clarified in the manuscript: ">60% sequence identity, the tantan repeat masking algorithm for alignments, and e-value ≤ 0.001."

4) Counts and coverage (p. 6): You state that "These 709 families captured 96.66% of the original unique sequence identifiers (103,385 out of 106,959).". However, a few lines above the final input set is reported as 169,605 unique protein sequences. Could you please clarify the initial number of sequences and the actual coverage after family generation and redundancy reduction?

Reply:
Thank you for catching this error. The number 169,605 resulted from a previous benchmark execution that was not then correctly updated. The correct numbers are 106,959 (116,959 including the additional UniProt sequences) and 5,826 produced seed clusters, not 2,947. The sequence coverage before family redundancy removal is 104,522 / 106,959 (97.72%), and 103,385 / 106,959 (96.66%) after redundancy removal. The manuscript has now been updated appropriately, and we apologise for this oversight.

5) Figures S1 and S2 are challenging to read due to low resolution.

Reply:
We thank the reviewer for pointing this out. The S1 and S2 figures have now been updated to high resolution.

em Editorial Manager®

Cookie Preference Center

We use cookies which are necessary to make our site work. We may also use additional cookies to analyze, improve and personalize our content and your digital experience. For more information, see our Cookie Policy.

You may choose not to allow some types of cookies. However, blocking some types may impact your experience of our site and the services we are able to offer. See the different category headings below to find out more or change your settings.

Allow all

Manage Consent Preferences

Strictly Necessary Cookies

Always active

These cookies are necessary for the website to function and cannot be switched off in our systems. They are usually only set in response to actions made by you which amount to a request for services, such as setting your privacy preferences, logging in or filling in forms. You can set your browser to block or alert you about these cookies, but some parts of the site will not then work.

Functional Cookies

These cookies enable the website to provide enhanced functionality and personalisation. They may be set by us or by third party providers whose services we have added to our pages. If you do not allow these cookies then some or all of these services may not function properly.

Performance Cookies

These cookies allow us to count visits and traffic sources so we can measure and improve the performance of our site. They help us to know which pages are the most and least popular and see how visitors move around the site.

Targeting Cookies

These cookies may be set through our site by our advertising partners. They may be used by those companies to build a profile of your interests and show you relevant adverts on other sites. If you do not allow these cookies, you will experience less targeted advertising.

Cookie List

Clear

Apply Cancel
Consent Leg.Interest

Confirm my choices

Powered by onetrust
