# Supplementary material for: nf-core/proteinfamilies: a scalable pipeline for the generation of protein families
Source: Gigascience. 2026 Jan 21;15:giag009. doi: 10.1093/gigascience/giag009 (PMC12950615; doi:10.1093/gigascience/giag009)
Supplement: giag009_Reviewer_1_Report_original_submission — 9/16/2025 [file giag009_reviewer_1_report_original_submission.pdf]

Reviewer Report

Title: nf-core/proteinfamilies: A scalable pipeline for the generation of protein families

Version: Original Submission **Date:** 9/16/2025

Reviewer name: Reviewer 1

Reviewer Comments to Author:

The authors present nf-core/proteinfamilies, a standardized Nextflow workflow that constructs protein families de novo or classifies sequences against existing families. Using a curated 200-family benchmark and a UniRef90-scale run, the authors show that the pipeline attains high recall with efficient runtimes. Given the ever-increasing size of sequence databases, this work is timely and fills a practical gap in reproducible, at-scale family curation; I expect it to be adopted widely by many research groups.I have several comments and suggestions below:1. In my view, this workflow will, by construction, yield a mixture of families: some anchored on a single conserved domain/segment, others centered on recurrent multi-domain cores, and some that capture the full-length sequence. This differs from widely used family databases: Pfam is largely domain-level, whereas HAMAP and NCBIFAM are mostly full-length/isofunctional (with PANTHER sitting in between). The resulting granularity is largely determined by MMseqs2 settings (sequence identity, query/target coverage, coverage mode) and by any alignment trimming, which biases toward conserved cores. Please add a brief discussion making this explicit, with practical guidance for tuning toward full-length versus domain-centric generation of families.I also recommend a parameter-sensitivity analysis on the 200-family set: sequence identity (30-70%), coverage thresholds (50-95%), and coverage mode (query/target/both), with and without trimming. For each setting, report (i) total families and split/merge rates per curated family, and (ii) a simple granularity readout, the proportion classified as domain-anchored, multi-domain, or full-length. This would clarify how parameter choices drive family counts and domain/full-length centricity, and help readers select defaults aligned with their use case.2. In the results, the splits/misses are concentrated in Pfam/PANTHER, while HAMAP/NCBIFAM are much closer to one-to-one (HAMAP 50/50). This suggests the inflated family count is driven, in part, by the domain-centric portion of the benchmark rather than the method itself. Please add a brief note in the Discussion to make this explicit.3. Since AFDB has models for most UniProt entries, could these models be used as an orthogonal purity check of the generated families; e.g., map members to AFDB and ask whether they cluster to the same fold by TM-score/Foldseek (allowing full-length differences when the family is domain-anchored)?4. HHsearch-based merging of divergent splits. In my view, and the authors note this, several curated families split simply because sequences are very divergent. An optional HHsearch (HMM-HMM) pass could merge these back: merge only at high probability (â‰ˆâ‰¥95%) with reciprocal coverage of the shorter model (â‰¥0.6). It would be useful to include this as an optional stage in the pipeline.5. Optional annotation of de novo families. I think it would be useful to add an annotation step that compares each de novo family (family HMM or MSA) against curated resources (Pfam, NCBIFAM, PANTHER/HAMAP).6. Could you briefly outline your expectations for how the pipeline handles transmembrane segments, coiled-coils,

repeats, and IDRs, classes prone to over-splitting under MMseqs2 seeding and trimming due to short-motif signal, low complexity, and variable lengths?

Level of Interest

Please indicate how interesting you found the manuscript: Choose an item.

Quality of Written English

Please indicate the quality of language in the manuscript: Choose an item.

Declaration of Competing Interests

Please complete a declaration of competing interests, considering the following questions:

- Have you in the past five years received reimbursements, fees, funding, or salary from an organisation that may in any way gain or lose financially from the publication of this manuscript, either now or in the future?
- Do you hold any stocks or shares in an organisation that may in any way gain or lose financially from the publication of this manuscript, either now or in the future?
- Do you hold or are you currently applying for any patents relating to the content of the manuscript?
- Have you received reimbursements, fees, funding, or salary from an organization that holds or has applied for patents relating to the content of the manuscript?
- Do you have any other financial competing interests?
- Do you have any non-financial competing interests in relation to this paper?

If you can answer no to all of the above, write 'I declare that I have no competing interests' below. If your reply is yes to any, please give details below.

I declare that I have no competing interests.

I agree to the open peer review policy of the journal. I understand that my name will be included on my report to the authors and, if the manuscript is accepted for publication, my named report including any attachments I upload will be posted on the website along with the authors' responses. I agree for my report to be made available under an Open Access Creative Commons CC-BY license (http://creativecommons.org/licenses/by/4.0/). I understand that any comments which I do not wish to be included in my named report can be included as confidential comments to the editors, which will not be published.

Choose an item.

To further support our reviewers, we have joined with Publons, where you can gain additional credit to further highlight your hard work (see: https://publons.com/journal/530/gigascience). On publication of this paper, your review will be automatically added to Publons, you can then choose whether or not to claim your Publons credit. I understand this statement.

Yes Choose an item.
