# Supplementary material for: nf-core/proteinfamilies: a scalable pipeline for the generation of protein families
Source: Gigascience. 2026 Jan 21;15:giag009. doi: 10.1093/gigascience/giag009 (PMC12950615; doi:10.1093/gigascience/giag009)
Supplement: giag009_Reviewer_2_Report_original_submission — 9/18/2025 [file giag009_reviewer_2_report_original_submission.pdf]

Reviewer Report

Title: nf-core/proteinfamilies: A scalable pipeline for the generation of protein families

Version: Original Submission **Date:** 9/18/2025

Reviewer name: Reviewer 2

Reviewer Comments to Author:

This manuscript presents a Nextflow pipeline (nf-core/proteinfamilies) for large-scale protein-family generation. Overall, I think the paper is well written and clear. The pipeline appears very useful, and the reported results show good performance in both family reproducibility and computational efficiency.I have a few minor comments requesting additional details:1) Does the quality-check step only compute statistics, or is it also used to filter/clean the input set? If so, please specify the criteria and whether filtered sequences are excluded downstream.2) Which MMseqs2 clustering mode is used (set cover, connected components, or greedy)? Can this be changed within the pipeline? If configurable, please indicate the relevant parameters.3) In the reproducibility benchmark, you use DIAMOND BLASTp to assess similarity between the initial sequence set for the selected families and additional Swiss-Prot sequences. Which sequence identity and alignment coverage (if any) thresholds were applied?4) Counts and coverage (p. 6): You state that "These 709 families captured 96.66% of the original unique sequence identifiers (103,385 out of 106,959).". However, a few lines above the final input set is reported as 169,605 unique protein sequences. Could you please clarify the initial number of sequences and the actual coverage after family generation and redundancy reduction?5) Figures S1 and S2 are difficult to read due to low resolution.

Level of Interest

Please indicate how interesting you found the manuscript: Choose an item.

Quality of Written English

Please indicate the quality of language in the manuscript: Choose an item.

Declaration of Competing Interests

Please complete a declaration of competing interests, considering the following questions:

- Have you in the past five years received reimbursements, fees, funding, or salary from an organisation that may in any way gain or lose financially from the publication of this manuscript, either now or in the future?
- Do you hold any stocks or shares in an organisation that may in any way gain or lose financially from the publication of this manuscript, either now or in the future?

- Do you hold or are you currently applying for any patents relating to the content of the manuscript?
- Have you received reimbursements, fees, funding, or salary from an organization that holds or has applied for patents relating to the content of the manuscript?
- Do you have any other financial competing interests?
- Do you have any non-financial competing interests in relation to this paper?

If you can answer no to all of the above, write 'I declare that I have no competing interests' below. If your reply is yes to any, please give details below.

I declare that I have no competing interests

I agree to the open peer review policy of the journal. I understand that my name will be included on my report to the authors and, if the manuscript is accepted for publication, my named report including any attachments I upload will be posted on the website along with the authors' responses. I agree for my report to be made available under an Open Access Creative Commons CC-BY license (http://creativecommons.org/licenses/by/4.0/). I understand that any comments which I do not wish to be included in my named report can be included as confidential comments to the editors, which will not be published.

Choose an item.

To further support our reviewers, we have joined with Publons, where you can gain additional credit to further highlight your hard work (see: https://publons.com/journal/530/gigascience). On publication of this paper, your review will be automatically added to Publons, you can then choose whether or not to claim your Publons credit. I understand this statement.

Yes Choose an item.
